# Affective–associative two-process theory: a neurocomputational account of partial reinforcement extinction effects

**DOI:** 10.1007/s00422-017-0730-1

**Published:** 2017-09-14

**Authors:** Robert Lowe, Alexander Almér, Erik Billing, Yulia Sandamirskaya, Christian Balkenius

**Affiliations:** 1 Department of Applied IT, University of Gothenburg, Gothenburg, Sweden; 2 Institutionen för informationsteknologi, Högskolan i Skövde, Skövde, Sweden; 3 Institute of Neuroinformatics, Neuroscience Center Zurich, University and ETH Zurich, Zurich, Switzerland; 4 Cognitive Science, Lund University, Lund, Sweden

**Keywords:** Partial reinforcement, Reinforcement learning, Decision making, Associative two-process theory, Affect

## Abstract

The partial reinforcement extinction effect (PREE) is an experimentally established phenomenon: behavioural response to a given stimulus is more persistent when previously inconsistently rewarded than when consistently rewarded. This phenomenon is, however, controversial in animal/human learning theory. Contradictory findings exist regarding when the PREE occurs. One body of research has found a within-subjects PREE, while another has found a within-subjects reversed PREE (RPREE). These opposing findings constitute what is considered the most important problem of PREE for theoreticians to explain. Here, we provide a neurocomputational account of the PREE, which helps to reconcile these seemingly contradictory findings of within-subjects experimental conditions. The performance of our model demonstrates how *omission* expectancy, learned according to low probability reward, comes to control response choice following discontinuation of reward presentation (extinction). We find that a PREE will occur when *multiple responses* become controlled by *omission expectation* in extinction, but not when *only one omission-mediated response is available*. Our model exploits the affective states of reward acquisition and reward omission expectancy in order to differentially classify stimuli and differentially mediate response choice. We demonstrate that stimulus–response (retrospective) and stimulus–expectation–response (prospective) routes are required to provide a necessary and sufficient explanation of the PREE versus RPREE data and that Omission representation is key for explaining the nonlinear nature of extinction data.

## Introduction

The *partial reinforcement extinction effect* (PREE) is characterized by a tendency for subjects to perseverate in behavioural responding to a greater degree when the behaviour was previously probabilistically/infrequently rewarded as compared to when it was unconditionally/frequently rewarded. These *partial*, as compared to *continuous*, schedules of reinforcement are critical for gaining insights into how a history of behaviour can bring to bear when circumstances change. Furthermore, intermittent reinforcement is the norm in *natural* environments (Pipkin and Vollmer 2009).

The PREE has been studied since the 1940s and 1950s (Mowrer and Jones 1945; Grosslight and Child 1947; Jenkins and Rigby 1950; Amsel 1958). It has been identified using a two-phase training assessment of behavioural history: (1) *an acquisition phase* where subjects are rewarded for engaging one of a number of response options in relation to a specific stimulus cue, (2) *an extinction phase* where subjects are no longer rewarded (or have diminished rewards) for responding. The PREE has been explained in terms of the *number of expected reinforcers omitted during extinction* (Gallistel and Gibbon 2000; Nevin 2012) so that multiple response choices in the extinction phase are required to be able to disconfirm probabilistic expectations learned in the acquisition phase. Thus, partial reinforcement (PRF) schedules require more responses than continuous reinforcement (CRF) schedules for such disconfirmation to be possible.

Nevertheless, controversies exist in the literature. The findings of a PREE given the above-mentioned comparison of CRF versus PRF schedules have been most consistently found in between-subjects investigations (Mowrer and Jones 1945; Grosslight and Child 1947; Svartdal 2008), i.e. when one set of subjects are tested on the CRF and a different set of subjects are tested on the PRF. The PREE has also been found using a within-subjects design (Kruse and Overmier 1982; Rescorla 1999; Nevin and Grace 2005a, b). However, within-subjects scenarios have also found a reversed PREE (RPREE) phenomenon. In this case, responding on the CRF schedule has actually been more resistant to extinction than the PRF schedule. The contradictory PREE and RPREE findings have been described as “[t]he outstanding difficulty” for PREE theory (Case 2000, p. 93).

### Theories for PREEs

There are several theories that attempt to address the underlying process of partial reinforcement effects on acquisition and extinction including those that attempt to address the contradictory PREE versus RPREE data, e.g. Nevin (1988); Nevin and Grace (2000) and behavioural momentum theory, and the sequential theory of Capaldi (1966, 1967, 1994). A subset of these theories provide mathematical models (Nevin 2012; Hochman and Erev 2013; Grossberg 1975, 2003).

One of the leading theories of the PREE is that of Amsel (1958, 1992), and is known as *frustration theory*. According to this theory, animals will work more vigorously (e.g. run down a maze faster) for a reward when they fail to receive anticipated reward, or when they anticipate non-reward, than when they receive, or anticipate, reward. The learned anticipatory frustration effect has been explained as the result of *dispositional memory*. This concerns a motivational effect of unexpected non-reward (e.g. increased arousal) on one trial being associated with a stimulus predictive of the reward that follows on the succeeding trial. Notwithstanding its explanatory power, it has been noted that within-subjects PREEs are not accounted for by this theory (Rescorla 1999).

*Associative Mediational Theory*, or AMT, (Trapold and Overmier 1972; Overmier and Lawry 1979; Kruse and Overmier 1982) is similar to frustration theory in that anticipation of reward omission can affect responding. Importantly, however, AMT states that conditioned expectancies can affect choice of responding, rather than just vigour or response rate. Different reward expectancies associated with different discriminative stimuli are then said to be used to mediate choice responses. The hypothesis made by Kruse and Overmier (1982) based on the AMT, was that *reward omission expectancy*, during the acquisition phase, should come to mediate behavioural responding (mediate discriminative choice) on a PRF schedule but not on a CRF schedule. However, during the extinction phase both an expectancy, and a response, switch should occur (Kruse and Overmier 1982) with omission expectation now controlling responses in both CRF and PRF conditions. Kruse and Overmier’s empirical results were consistent with their AMT hypothesis.

Svartdal (2008)—following up on human subject experiments described in Svartdal (2000)—posited a *modulation hypothesis* to explain his findings of (1) between-subjects conventional PREE, and (2) within-subjects RPREE. The modulation hypothesis claims that use of different, alternating, reinforcement schedule components in within-subjects experiments modulates behaviour in relation to extinction resistance: the higher reinforcement probability component modulates resistance of the lower probability component *downwards*, i.e. it lessens resistance; the lower reinforcement probability schedule modulates resistance of the higher probability component *upwards*, i.e. it increases resistance. This modulation upwards or downwards is in relation to the single (between-subjects) reinforcement schedules (i.e always low reward probability, or always high reward probability). The mechanism underlying this modulation is, however, unclear.

Models concerning the neurobiology of partial reinforcement extinction effects are surprisingly lacking. Notwithstanding, there is evidence for separate representations of both reward- and omission-based expectation in the brain of animals and humans. Watanabe et al. (2007) describe the finding of neural activity in the orbitofrontal cortex (OFC) correlating with omission of expected reward during a delay period (from predictive cue onset to the time at which reward is intermittently delivered). McDannald et al. (2005) have suggested that it is the interaction between the OFC and the basolateral component of the amygdala (BLA) that is responsible for the encoding of reward and omission expectations associated with the eliciting primary stimuli and responses. The interplay between OFC and BLA has been said to be at the heart of affective or emotional appraisal of reward (acquisition) and (reward) omission preceding the elicitation of particular emotions/affective states, including excitement, and frustration, respectively (Rolls 1999, 2013). Medial prefrontal cortex (Passingham and Wise 2012), and dorsolateral prefrontal cortex (Watanabe et al. 2007) have been suggested to have respective roles in outcome-contingent learning and choice, and integration of retrospective and prospective memory that may amount to a sort of competition mediating response choice.

### Aims of the study

In this article, we propose a neural-computational account of the partial reinforcement extinction effect (PREE). We put forward our affective–associative two-process theory to model the PREE. ATP theory (Trapold and Overmier 1972; Urcuioli 2005) extends associative mediational theory (Kruse and Overmier 1982; Overmier and Lawry 1979) as an associative learning account of differential outcome learning phenomena such as the PREE. In using an associative explanation of the learning process, we comply with the default position in animal learning theory (Pearce 2006), i.e. avoiding recourse to extraneous cognitive mechanisms in preference for the conceptually simplest explanation. Using our model, we simulate the results of two studies whose experimental set-ups are comparable. This comparison is of interest because in spite of the similarity of the set-ups used, contradictory results were found (Kruse and Overmier 1982; Svartdal 2008). Our modelling approach affords a re-visiting of ATP theory offering new perspectives (cf. Urcuioli 2005, 2008, 2013) regarding the role of affective states in stimulus classification. The aims of our modelling approach are: (1) show that the model, implemented as a neurocomputational circuit, can capture the contradictory PREE and RPREE findings of the two studies; (2) describe the mechanism that underlies the *modulation effect* of Svartdal and the non-modulation effect of the Kruse and Overmier (1982) experimental set-up; (3) describe and demonstrate the model’s capability of accounting for existing PREE theory as well as other related learning phenomena.

This article breaks down as follows: In Sect. 2, we describe our model, explaining how omission anticipation representations can be learned and serve to mediate response selection. In this methodology section we also describe the nature of our simulations of the two experiments under investigation. In Sect. 3, we simulate the Kruse and Overmier (1982) and the Svartdal (2008) findings using our computational model. In Sect. 4 we show how the empirical data can be explained in terms of ‘stimulus classification by outcomes’ (Urcuioli 2013); we validate our model using a ‘lesioning’ approach and carry out a parameter sensitivity analysis to show the range of learning rates within which our model is operational. Finally, in Sect. 5, we provide a general discussion regarding the plausibility of this model and predictions it makes.

## Methodology: simulation set-ups and modelling approach

### Simulation set-ups: Kruse and Overmier (1982) versus Svartdal (2008)

The Kruse and Overmier (1982), experiment 1, and Svartdal (2008) experiment were chosen for our neural-computational analysis as suitable examples of the contradicting within-subjects PREE versus RPREE bodies of research. Kruse and Overmier obtained a PREE while Svartdal obtained a RPREE. The experiments used rat and human subjects, respectively, providing one potentially significant source of the difference in the results. However, the PREE and RPREE have been found in rats and humans (as well as other animals) and evidence for the existence of cross-species associative mediational processes obtained through differential outcomes training procedures is well-documented (Urcuioli 2005).

**Fig. 1:**
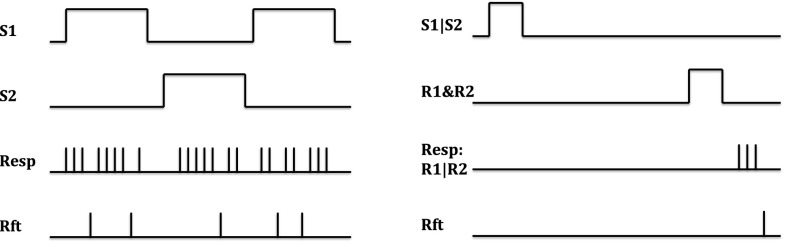
Common experimental set-ups for evaluating partial reinforcement extinction effects. *Left* Delay conditioning with free operant responding—subjects, typically non-human, are evaluated according to response rate in the presence of discriminative stimuli. The stimuli inform of the variable interval schedules of reinforcement (adapted from Nevin and Grace 2000). *Right* Trace conditioning with differential responding—subjects are evaluated according to choice correctness in the presence of discriminative stimuli. The stimuli are briefly presented followed by an inter-stimulus interval (ISI). Response options are presented at the end of this interval and following a response (multiple responses may be required) a reinforcer is received. The trial is terminated (discrete trial) and followed by an inter-trial interval (ITI) Key: *S1/S2* stimulus 1/2, *Resp* response, *R1/R2* response options 1/2, *Rft* reinforcement

We focus on these two particular experiments owing to their methodological similarity. Firstly, they both involve the potential to associate discriminative stimuli with one of multiple instrumental responses. Secondly, they both utilize discrete learning trials for making evaluations of choice correctness history (based on reward-relevant feedback). Thirdly, they use a trace conditioning set-up (Fig. 1, right): discriminative stimuli are briefly presented then withdrawn, a pause follows, then response options are presented, and then a rewarding outcome is presented. The reader is referred to “Appendix 6.1” for a summary of the two experimental set-ups (as compared to the respective simulated set-ups). Many investigations of the PREE use instead delay conditioning set-ups (Nevin and Grace 2000) whereby stimuli, responses and reinforcers may temporally overlap (see Fig. 1 for comparison of set-ups). In sum, similarity in experimental set-ups permits relative ease of analysis in our simulations set-up.

**Fig. 2:**
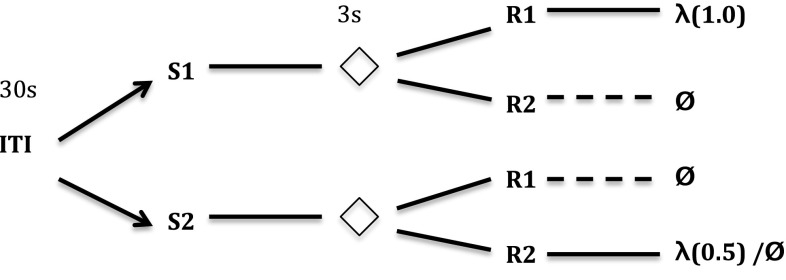
Kruse and Overmier (1982) schema of procedure. *ITI* inter-trial interval, *S1/S2* stimulus 1/stimulus 2 (sonalert, clicker stimuli order of presentation per trial varied per subject); $\Diamond$ represents pause period; *R1/R2* response 1/response 2 (lever presses to the left or right of the food panel); $\lambda$ indicates the reward (food pellets) with probabilities of receiving food given in brackets; $\phi$ indicates no reward. One stimulus is presented at random per trial, one response type (R1 or R2) is permissible per trial. The *dashed lines* here indicate the ‘incorrect’ response option

Kruse and Overmier’s set-up required individual rats to perform in a Coulborn operant chamber. The rats, over a number of discrete learning trials, were firstly presented with one of two sensory stimuli. Following a short (3 s) delay, levers were presented that flanked the centrally located food collection box. Once a criterion number of successive correct lever presses (ten) were produced by the rat (i.e. above baseline pressing), a reward (food pellets) was presented in the food collection box. The correct response (lever press) choice was rewarded according to a probabilistic schedule (1.0 versus 0.5 probabilities) depending on the experimental condition (between subjects) or trial (within subjects), i.e. continuous, or partial, reinforcement conditions, respectively. In the case that the incorrect response was made, no reward was forthcoming. This experimental sequence is illustrated in Fig. 2 as the within-subjects CRF versus PRF multiple-schedule (experimental) condition. In this case, the two reward discriminating stimuli (clicker, sonalert tone) differentially signalled the reinforcement schedules, continuous (CRF) or partial (PRF). For one discriminative stimulus (S1), one response was required (R1); for the other discriminative stimulus (S2), the other response was required (R2). In the between-subjects conditions, the same schedule (always CRF or always PRF) was used in relation to the rewarding S–R contingencies.

**Fig. 3:**
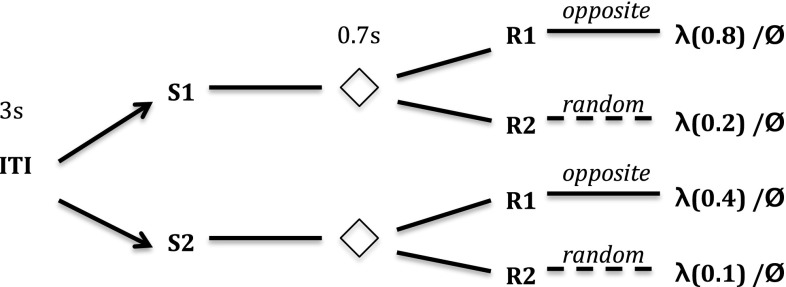
Svartdal (2008) schema of procedure. *ITI* inter-trial interval; *S1/S2* stimulus 1/stimulus 2 (red, green lights varied per subject); $\Diamond$ represents pause period; *R1/R2* response 1 (produce opposite button press sequence to computer), response 2 (produce random sequence); $\lambda$ indicates the reward (correct choice feedback) with probability of receiving feedback given in brackets; $\phi$ indicates no reward. One stimulus is presented at random per trial, one response is permissible per trial. *Dashed lines* are for ‘incorrect’ choice

The experiment of Svartdal (2008), by contrast, involved human subjects, who were required to press buttons in response to a two-button sequence presented (through an automated computer program) as feedback on a computer monitor on a table at which they were seated. Following presentation of one of two colours on the screen (the discriminative stimuli), the sequence was presented and, following a short delay (0.7 s), the subjects were then required to respond to the computerized sequence using the left and right buttons in front of them. Correct choice entailed learning that the subject’s button presses were required to be the opposite of that presented on the screen in order to get the feedback of being ‘correct’ (the effective reward). In this case, subjects were presented one of two discriminative stimuli (S1 or S2), but the same response (R1; produce opposite response sequence to the computer program) was necessary to make the correct choice for both stimuli. The alternative response options were non-rewarding. In effect, the response options were: R1—choose opposite response sequence to the computer program, R2—choose a response based on an incorrect, e.g. random, strategy. For the sake of our simulations, we assume that the simplest, i.e. random, strategy is most likely applied by subjects prior to inferring the task rules. The Svartdal 2008 experiment is illustrated in Fig. 3.

In effect, S–R links or associations entail the learning of rules. In Kruse and Overmier (1982) this manifests in learning that S1–R1 and S2–R2 are rewarding, whereas other combinations are not, while in Svartdal (2008) S1–R1 and S2–R1 are rewarding while other combinations are not. In this case, R must be considered as a response choice, which in the case of Kruse and Overmier (1982) concerns pressing one or other lever, whereas in Svartdal (2008) it concerns applying the button presses that are opposite to those presented on the computer monitor.

### A neural-computational model of affective–associative two-process theory

**Fig. 4:**
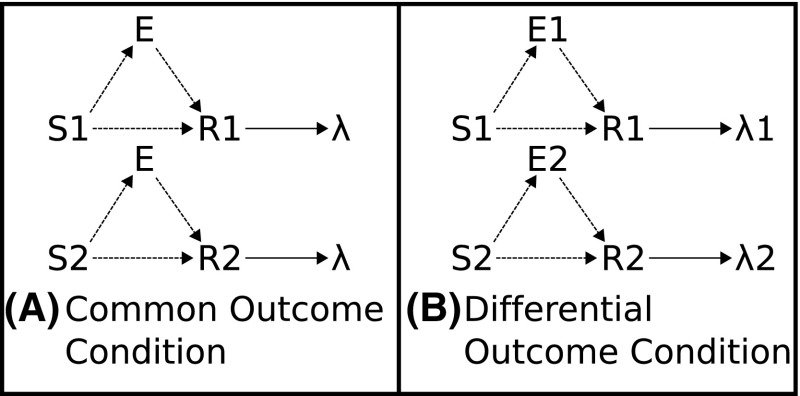
Associative two-process theory and the differential outcomes effect. **A** Common outcome condition. Reinforced S–R associations cannot be distinguished by outcome. **B** Differential outcome condition. Reinforced S–R associations can be distinguised, and cued, by differential outcome expectancies (E1, E2)

Associative two-process (ATP) theory provides a strong candidate to explain the workings of the PREE and is considered the leading theoretical explanation of the differential outcomes effect (DOE) (Trapold 1970; Urcuioli 2005, 2013). ATP hypothesizes the formation, during learning, of associations between stimulus (S) and expectations of outcome (E) and in turn of E–R (responses) associations. These associations, thereby, provide an alternative, ‘prospective’, route to response selection to the traditional instrumental S–R route. This relationship is captured in Fig. 4.

In the Kruse and Overmier (1982) experimental set-up, the difference between the stimulus predicting a reward with 0.5 probability as compared to the stimulus predicting reward with probability of 1.0 “allowed the mediating internal expectancy state, presumably anticipatory frustration in this case, to gain at least partial control over one response, while the expectancy of reward exercised full control over the other” (Kruse and Overmier 1982).

#### Neurophysiological correlates of affective–associative computation

**Fig. 5:**
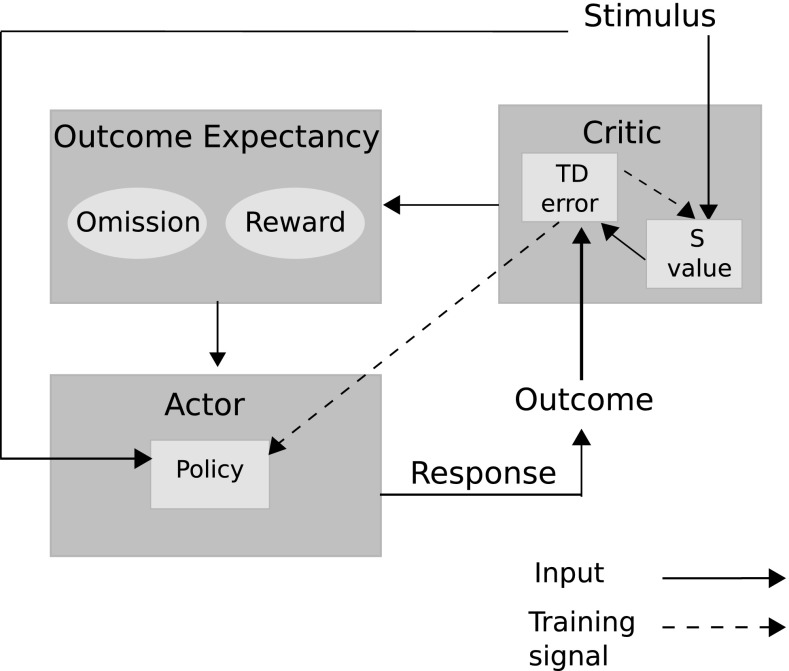
Affective–associative two-process model. The model is a hybrid of an actor–critic architecture embedding within an associative mediational theoretic (AMT) component (‘outcome expectancy’). Traditional actor–critic architectures are linked through the temporal difference (TD) prediction error that updates both the stimulus valuation (S value) and the action valuations (policy). In the associative–affective two-process model, the AMT component further links critic to actor through learned connections. This provides an alternative route, to the stimulus–actor (response) route for action selection. The affective component concerns omission and reward expectations, following specific stimulus presentations, that become associated with responses

We hypothesize that affective–associative circuitry in the brain takes on an actor–critic-like structure where the critic computes dimensions of value of discriminative stimuli, and the actor utilizes values as expectancies for mediating response choice (see Fig. 5). Such actor–critic networks have been hypothesized to exist as implementing interactions between basal ganglia (actor-like) and cortical structures (critic-like), e.g. Houk and Adams (1995). Many other cortical actor–critic components have been proposed (Silvetti et al. 2014) including medial PFC (Silvetti et al. 2014) and dorsal PFC (Holroyd and Yeung 2012) for actor-like structures as well as orbitofrontal cortex (OFC), (Holroyd and Yeung 2012) and anterior cingulate cortex (ACC) (Silvetti et al. 2014), for critic-like structures. A restriction on a standard actor–critic network for modelling affective–associative circuitry is that value (expectancy) computations cannot directly mediate responses, in the manner that the ATP requires. Instead, error signals that result from unpredicted reinforcer presentations to the network, are used to learn associations between the discriminative stimuli and the responses that led to these errors (updating a ‘policy’). This is consistent with classical two-process theory, which focuses on a S–R processing route.

The ‘critic’, in our modelling approach, implements a method for computing outcome expectancies. Our model builds on that of Balkenius and Morén (2001) which computes two dimensions of value within its critic-like component. These dimensions correspond to reward omission probability and reward magnitude. In Balkenius and Morén (2001)—and Morén (2002)—the omission network and magnitude network were considered to implement computational processes that can be found in the OFC and amygdala, respectively. The OFC is considered to enable fast, flexible and context-based learning (particularly important in studies of reversal learning, e.g. Delameter 2007) whereas the amygdala is considered less flexible, i.e. resistant to unlearning, but critical to learning valuations of stimuli (Schoenbaum et al. 2007). Furthermore, the interplay between the basolateral division of the amygdala (BLA) and OFC may be crucial in differential reward evaluation (Ramirez and Savage 2007). The actor for our model is required to utilize the differential outcome expectancies to mediate choice responses. Passingham and Wise (2012) have suggested that medial prefrontal cortex (PFC) has a critical role in encoding outcome-contingent choice, whereas Watanabe et al. (2007) have provided evidence for the lateral PFC integrating activation inputs from ‘retrospective’ (working memory) areas such as dorsal PFC and ‘prospective’ (outcome expectant) areas such as OFC and medial PFC.

**Fig. 6:**
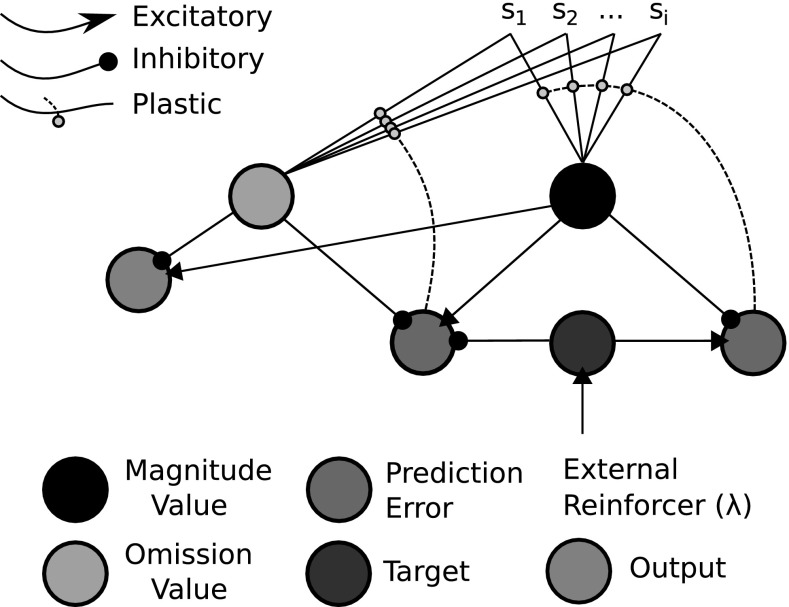
Balkenius and Morén (2001) model. The model adapts the Rescorla and Wagner (1972) scalar reinforcement function to allow for two dimensions of value—an effective reinforcer magnitude function, and an effective reward omission function. See also Morén (2002) for details

**Fig. 7:**
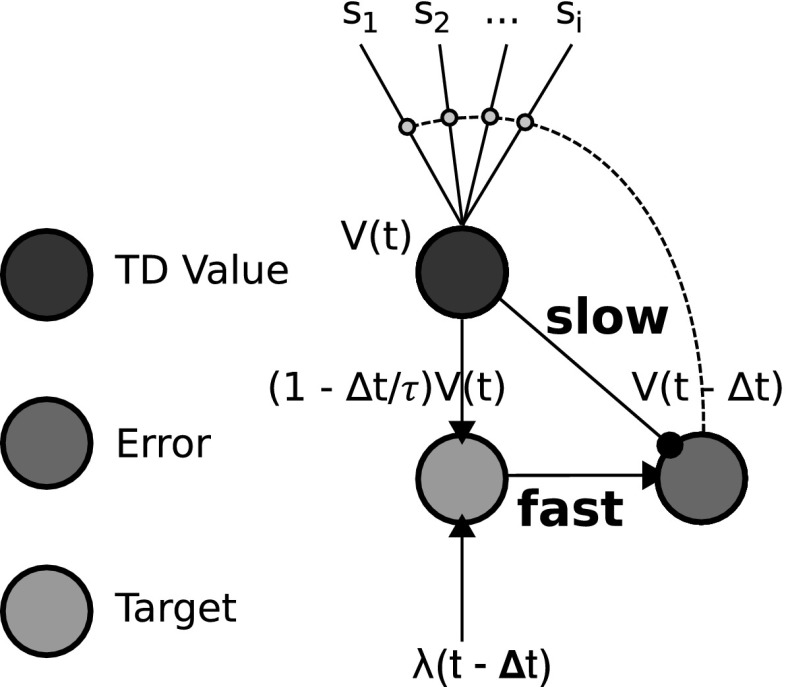
Neural-computational temporal difference learning algorithm (adapted from Trappenberg 2010). For a neural computational implementation to be faithful to the TD learning algorithm, the assumption that there are fast and slow connectivity routes to the prediction error node is required. The computations of the individual nodes reflect the continuous time Doya (2000) implementation of TD learning—see Eq. (5) in main text

#### Computational derivation of the model

At the root of the affective–associative component of our model is the temporal difference learning algorithm of Sutton and Barto (1990, 1998). This algorithm conflates into a single dimension of value the information about multiple reinforcement properties of the stimulus. In animal learning, the use of a scalar value function has been noted as a key limitation of the Rescorla–Wagner model (Miller et al. 1995). As an example of its limitation, a reinforcer magnitude of 1.0 and presentation probability 0.5 is valued equivalently to one of magnitude 0.5 and presentation probability 1.0. Organisms may, nevertheless, benefit from multi-dimensional reinforcer information. For example, high magnitude, low probability reinforcers might motivate learning the causal antecedents of the low presentation probability so as to increase future reward yield (Mackintosh 1971) and actively reduce prediction error (Pezzulo et al. 2015).

Balkenius and Morén (2001)—see also Morén (2002), Balkenius et al. (2009)—presented a model of learning (Fig. 6) that addresses the above-mentioned limitation by deriving a computation of reinforcement omission from a reinforcement magnitude computation. Although not explicitly noted by the authors, this effectively provides an omission probability when taken as a fraction of the reinforcement magnitude. For every trial a reward is not presented to the network, the error node of the omission critic is disinhibited. Omission error then receives input from the Magnitude value node with which it updates its own omission (probability) representation. With repeated trials, the omission probability is more accurately approximated (learned). It updates asymptotically as increasingly accurate omission probability (value) leads to increasingly inhibited error signals. The ‘probability’ computation is only possible because the magnitude component only learns the value of a reinforcer over trials but *does not unlearn* in its absence. It thus provides an accurate measure of magnitude given that learned magnitude does not vary over trials.

The Balkenius and Morén architecture is, however, limited by its incapacity to represent time. This is a critical feature for models that attempt to capture neurobiologically realistic activation patterns and the discounted valuation effect of *duration* of the inter-stimulus interval^1^ on action selection. The temporal difference learning algorithm of Sutton and Barto (1990, 1998) addresses this limitation. It has been viewed as being at the intersection between animal learning and machine learning investigations (Wörgötter and Porr 2005).

**Fig. 8:**
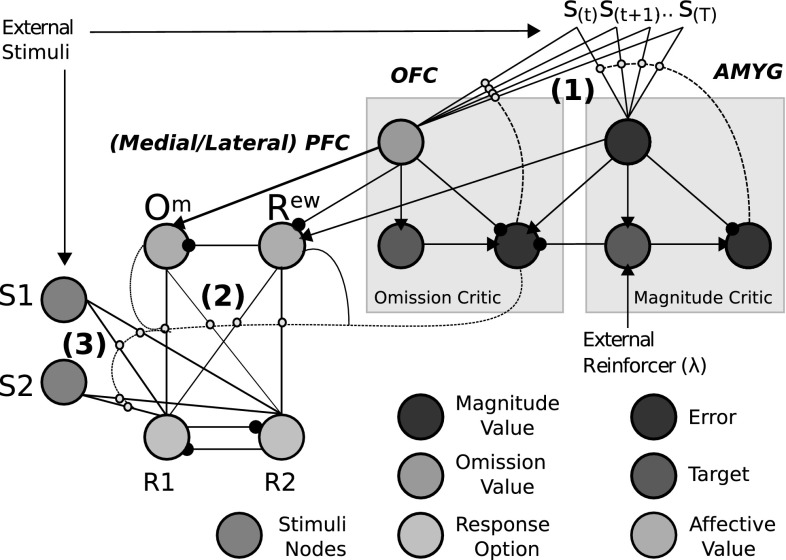
Affective–associative two-process network. Our associative–affective component builds on Trappenberg’s and Balkenius and Morén’s (2001) neural network models. It further adds an ‘actor’ whereby the outputs of the two ‘critic’ value dimensions become associated with response options. Mutual inhibition promotes dimensional mediation of responses. The computations of individuals nodes are derivable according to Fig. 7 and Eqs. (1–12). *Small yellow circles* indicate learnable connections (gated by prediction error value). S1/S2 connections to R1/R2 provide the ‘retrospective’ (S–R; label 3) route of ATP. The Om and Rew connections to R1/R2 provide E–R connections (2). Finally the S(t) inputs here provide temporal stimulus valuations and allow for S–E connections (1). Key: *OFC* orbitofrontal cortex, *AMYG* amygdala, *PFC* prefrontal cortex (colour figure online)

Figure 7 shows a neural network implementation of the TD learning rule (adapted from Trappenberg 2010). While neural network TD models have previously existed (Suri and Schultz 1998; Balkenius and Morén 1999; Suri 2002), the depiction of Trappenberg (2010) shows the requirement for fast and slow connections between a value computation node and two other computational nodes. The link between a target^2^ node (in green) and the value node (in black) constitutes the difference in computation of a TD learning network and a Rescorla–Wagner learning network. In Fig. 6 (the Balkenius and Morén 2001 model) the absence/presence of this link, similarly, constitutes a key difference in critic computation between our model (Fig. 8) and the Balkenius and Morén (2001) model (Fig. 6).

From the Trappenberg (Fig. 7) and Balkenius and Morén (Fig. 6) neural network models, we have derived our affective–associative model which is shown in Fig. 8—see Lowe et al. (2014) for an earlier version. Corresponding to the numbered elements in the figure, it consists of (1) *S-E learning*: A value function (critic) computing reward omission probability and magnitude, which is a TD learning adaptation of the Balkenius and Morén (2001) model, (2) *E-R learning*: An expectancy behaviour mediation network, which extends the Balkenius and Morén (2001) model’s expectation-based behavioural control by allowing for multi-response associative mediation, (3) *S-R learning*: A stimulus–response associative route.

In (1), omission critic computation can be explained as follows:

- *Learning omission expectation* the omission critic error node (Fig. 8) updates omission value when reinforcement via the magnitude critic ‘Target’ node is absent at the previously learned time.
- *Asymptotic learning* Omission expectation inhibits the omission error as an asymptotic and temporally discounted function of existing expectation.
- *Unlearning* Above-zero omission expectation decreases as a result of unexpected reinforcement input.

In the case of (2), like Balkenius and Morén (2001), we have the output of the omission critic inhibit the output of the magnitude critic (at the ‘Rew’ node). This preserves the ability of the network to account for the empirical data that the Balkenius and Morén model simulated, e.g. asymptotic profiles of learning and extinction, and the ‘savings effect’ (Pearce 2006)^3^—see Lowe et al. (2014) for data. The savings effect is achieved by Balkenius and Morén (2001) by having differential learning rates for omission, and magnitude, computations, which is made possible by separating the standard value function into these two dimensions. The output node of the Balkenius and Morén (2001) model constitutes an ‘optimistic’ (reward acquisition) probability value. The magnitude value is equivalent to the maximum probability in this case as omission probability is taken as a fraction of it. In our model, the node ‘Rew’ implements the same function.

Additionally, we model omission representation (‘Om’). This ‘pessimistic’ (omission) probability representation receives inhibition from the ‘Rew’ node. Both ‘Om’ and ‘Rew’ nodes, however, receive nonlinear transformations of inputs—see Eqs. (9–11). Thus, they no longer accurately represent probabilities but rather more general expectations of reward acquisition (‘Rew’) and frustrative non-reward, or omission (‘Om’). A glossary of key terms linking aspects of our model in Fig. 8 to the equations in the following subsections is found in “Appendix 6.2”.

#### Critic equations

Equations (1–6) provide the implementation of the critic. In simulation, for each trial, one cue input to the critic is presented at time $t=25$ and offset at $t=50$, the two target inputs (at S1 and S2 nodes, respectively) are presented at $t=57$ and offset at $t=72$, during which time a response (above threshold) is chosen, and reinforcer input (or not) is presented at $t=72$, and offset at $t=73$. The inter-stimulus interval (ISI) between stimulus and target(s) is, thus, 7 timesteps and ISI between stimulus and reinforcer is 22 timesteps.

1 $$\begin{aligned} V_{e}(t)= & {} f^{\prime }\left( \sum _{n=1}^{N} \sum _{s=1}^{S} \left( \theta _{e_{ns}}(t)\phi _{ns}(t)\right) \right) , \end{aligned}$$

2 $$\begin{aligned} f^{\prime }(x)= & {} \left\{ \begin{array}{l l} 0, &{} \quad x<0\\ x, &{} \quad x \ge 0 \,\text {and}\, x <1\\ 1, &{} \quad x \ge 1 \end{array} \right. \ \end{aligned}$$

3 $$\begin{aligned} \theta _{e_{ns}}(t)= & {} \theta _{e_{ns}}(t-\Delta t) + \beta _e \delta _e(t) \bar{e}_{n}(t) \end{aligned}$$

4 $$\begin{aligned} \bar{e}_{n}(t)= & {} \left\{ \begin{array}{l l} 1, &{} \quad t < t0\\ \lambda \gamma \bar{e}_{n}(t-\Delta t), &{} \quad t \ge t0 \end{array} \right. \ \end{aligned}$$

where $V_{e}(t)$ is the learned value function (expectation); $\theta _e(t)$ is the value function update rule that, through prediction error updating, valuates the temporal stimuli; *e* $\epsilon$ $\{m,o\}$ is an index denoting Magnitude or Omission critic value functions, respectively; *n* is the number of stimuli discrete trace representations in [1, *N*] where $N=100$; *t* is time in [1, *T*] where $T=100$; *t*0 $=$ the time onset of reward; *s* is the number of different stimuli in [1, *S*] where $S=2$; $\beta _e$ is a learning rate in [0,1); $\Delta t$ is the time window set here to 1; $\delta _e$ is the prediction error term (non-negative for $e=m$); $\phi$ is the input stimulus vector of size $=$ [100, S] (for each stimulus in S there is a vector of 100 timesteps). This temporal stimulus representation formulation is known as the complete serial compound (CSC), as used by Suri and Schultz (1998). Each vector of the compound stimulus has a single unit set to 1 and all others set to 0. A given vector represents the trace delay of a phasically (short duration) presented stimulus across the inter-stimulus interval (time between stimulus and reinforcement presentations). This means a unique vector represents a time step following offset of the stimulus presentation whose unity value provides a pre-synaptic component of the two-factor learning rule (the other ‘factor’ being the prediction error). Equation (3) provides the value function update rule that associates the stimuli with the prediction error term via an eligibility trace (calculated in Eq. 4), following Doya (2000). Equation (4) provides the backward view TD($\lambda$) implementation of the eligibility trace used to speed up learning—each temporal stimulus representation unit in the CSC has an eligibility trace following onset (set to 1) that decays at rate $\lambda \gamma \bar{e}_{n}(t-\Delta t)$, where $\lambda =1-\frac{1-\Delta t/\kappa }{1-\Delta t/\tau }$ ($\kappa =9.6$, $\tau =10$) and $\gamma =1-\frac{\Delta t}{\tau }$, following Doya (2000).

5 $$\begin{aligned} \delta _{m}(t)= & {} \lambda (t - \Delta t) + \frac{\tau }{\Delta t}\left( \left( 1-\frac{\Delta t}{\tau }\right) V_m(t) - V_m(t-\Delta t)\right) \end{aligned}$$

6 $$\begin{aligned} \delta _{o}(t)= & {} -\delta _{m}(t) + \frac{\tau }{\Delta t}\left( \left( 1-\frac{\Delta t}{\tau }\right) V_o(t) - V_o(t - \Delta t)\right) \end{aligned}$$

where $\delta _{m}$ and $\delta _{o}$ represent prediction errors used to update the magnitude and omission critics, respectively, and to approximate them better as Bellman optimality functions; $\lambda (t)$ is the reward signal in [0, 1]; $\tau$ is a time constant.^4^ We hereby use a set of parameters for the critic that is based on theoretical considerations of reinforcement learning.

#### Actor equations

The nodes (S1, S2, R1, R2: see Fig. 8) in the actor network are governed by the neural-dynamic Eqs. (7) and (8). Equations (9–11) provide Rew and Om node parameterizations (again see Fig. 8).

7 $$\begin{aligned} \begin{aligned} u_r(t)&= u_r(t-\Delta t) + \frac{\Delta t}{\tau _r}\left( -u_r(t-\Delta t) \right. \\&\quad \left. +\,h_r + C_r \varLambda (u(t-\Delta t,\beta _r)) + I_r(t)\right) \end{aligned} \end{aligned}$$

where $u_r(t)$ provides the backward Euler differentiation description of Amari (1977) nodes (only 1 node for each *r*) and represents the activation of the *r*th node $r \in [1,R]$ and $R = 4 (1=S1, 2=S2, 3=R1, 4=R2)$; $C_r \varLambda (u(t-\Delta t,\beta _r))$ provides self-excitation scaled by $C_r$; $I_r(t)$ is the input term. Free parameters are listed in “Appendix 6.3”.

8 $$\begin{aligned} \varLambda (u,\beta ,th) = \frac{1}{1 + \exp [-\beta (u-th)]} \end{aligned}$$

$\varLambda (u,\beta ,th)$ provides a nonlinear (sigmoidal) transformation of activation for all actor nodes where $\beta$ provides a gain parameter and *th* a threshold value.

9 $$\begin{aligned} {\hbox {Rew}}(t)&= \varLambda \left( \left( V_m(t-1),x_{\beta _\mathrm{vm}}(t),x_{th_\mathrm{vm}}(t)\right) \right. \nonumber \\&\left. \quad -\,\varLambda \left( V_o(t-1),x_{\beta _\mathrm{vo}}(t),x_{th_\mathrm{vo}}(t)\right) \right) \end{aligned}$$

10 $$\begin{aligned} Om(t)&= \varLambda \left( \varLambda \left( V_o(t-1),x_{\beta _\mathrm{vo}}(t),x_{th_\mathrm{vo}}(t)\right) \right. \nonumber \\&\quad \left. -\,Rew(t),\beta _\mathrm{om},th_\mathrm{om}\right) \end{aligned}$$

11 $$\begin{aligned} x_{s}(t)&= \left\{ \begin{array}{l l} n_{s}, &{} \quad x_{s}<n_{s}\\ a_{s}, &{} \quad x_{s}\ge a_{s}\\ x_{s}(t-\Delta t)+\psi _{s} \cdot \delta _{o}\cdot \frac{1}{C_j\cdot I_r(t)}, &{} \quad {\hbox {otherwise}} \end{array} \right. \ \end{aligned}$$

Equations (9–11) concern Rew and Om node activations and meta-parameterization that permits a sort of ‘classification by stimulus’ Urcuioli (2013) where $\beta _\mathrm{om}$, $th_\mathrm{om}$ provide sigmoid inputs. $x_s$ parameterizes the sigmoid update functions for (Om, Rew nodes) according to slope $x_{\beta _s}(t)$, $s=\{$1(Om),2(Rew)$\}$ and threshold $x_{th_s}(t)$, $s=\{$3(Om),4(Rew)$\}$. These are meta-parameters (Doya 2002) modulated by the prediction error feedback of the omission critic.

The parameter $\psi _{s}$ gives a binary sign value. $\psi _{s}$ is positive for $\beta$ and negative for *th* ($V_o$ inputs) so that the omission critic prediction error increases the gain on the slope and brings towards zero the threshold of the *omission* node sigmoid function. $\psi _r$ is negative for $\beta$ and positive for *th* ($V_m$ inputs) so that the omission critic reward prediction error (inverted omission critic prediction error) increases the gain on the slope and brings towards zero the threshold of the *reward* node classifier. Finally, $C_j$ concerns the meta-learning rate. The stronger the positive omission prediction error, the larger the possibility for effective omission classification; the stronger the positive reward prediction error, the greater the tendency for effective reward classification. Example sigmoid functions for Rew and Om are shown in “Appendix 6.4” (Fig. 23). The use of dopamine (prediction error) to increase precision and confidence in signalling has been hypothesized by predictive processing accounts (Friston et al. 2012; Clark 2015; Pezzulo et al. 2015).

12 $$\begin{aligned} \begin{aligned}&\varOmega _{kl}(t) = \varOmega _{kl}(t-\Delta t) \\&\qquad \qquad +\,\beta _e \delta _e(t) \varLambda \left( u_k(t),\beta _r\right) \varLambda \left( u_l(t),\beta _r\right) \end{aligned} \end{aligned}$$

$\varOmega _{kl}(t)$ provides connectivity strengths (synaptic efficiencies) from pre-synaptic node $k\in \{Om,Rew\}$ to post-synaptic node $l\in \{R1,R2\}$.

See Sect. 4 for discussion of learning rates $\beta _e$ where *e* $\epsilon$ [S-R,S-$V_m$,S-$V_o$,E-R].

## Results

### Simulation of Kruse and Overmier (1982) within-subjects experiment

Typical to partial reinforcement extinction investigations, there were two phases in the Kruse and Overmier (1982) experiments: *acquisition phase*, for learning reinforcement value; *extinction phase*, where reinforcers are no longer presented. There were three conditions: (1) Experimental (EXPL)—a within-subjects design where a given reinforcer schedule (continuous/CRF or partial/PRF) was randomly selected per trial^5^ (see Fig. 2), (2) CRF between-subjects control, i.e. both components were continuous, (3) PRF between-subjects control, i.e. both schedules were partial. Where in (1) stimulus 1 is reinforced by R1 (CRF) and stimulus 2 reinforced by R2 (PRF), in (2) and (3) the two stimulus–response contingencies now lead to purely CRF, or PRF-based outcomes, respectively.

#### Acquisition phase

**Fig. 9:**
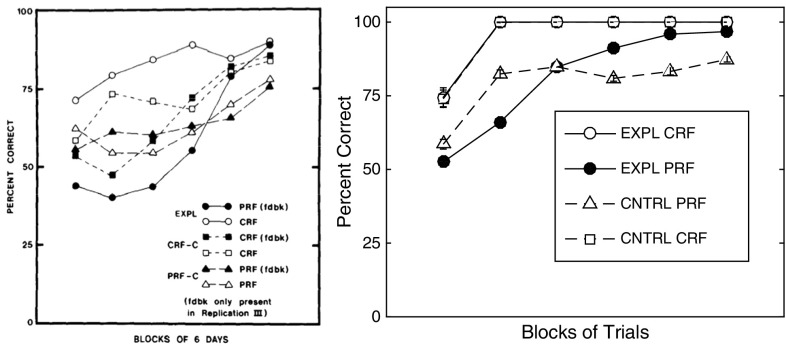
Comparison of the present theoretical predictions with Kruse and Overmier acquisition results. *Left* original experimental results due to Kruse and Overmier (1982), reprinted with permission. *Right* simulated results. The six data points (*right*) concern blocks of 40 trials each

The results of the acquisition phase are displayed in Fig. 9.

In Fig. 9 (left) the empirical data is shown (from Kruse and Overmier 1982) of mean values of correct behavioural choice, i.e. reinforced according to one or other schedule (PRF or CRF) for each condition. Here it can be seen that in the EXPL condition the CRF trials lead to faster learning (acquisition) than do the PRF trials though the latter converges onto the former’s performance (near 90% correct response choice) by the sixth day of trials. The between-subjects CRF (non-feedback) and PRF (non-feedback) conditions^6^ fair observably better and worse, with respect to their within-subjects counterparts (in the EXPL condition). The result of increased acquisition speed under CRF conditions is expected under the associative mediation theory (AMT) of Kruse and Overmier (1982), but also most theories concerned with partial reinforcement effects, since the higher rate of reinforcement leads to more rapidly acquired associations between predictive (conditioned) stimuli and response options. This within-subject difference in acquisition speed was found by the authors to be statistically significant.

Our simulation results are displayed in Fig. 9 (right). They show the mean values^7^ of correct choice response. In this case, the acquisition rates of each of the three conditions (EXPL, CRF, PRF) are shown where similar findings were obtained as compared to the empirical data. In this case, we did not have additional ‘feedback’ conditions and, therefore, here show instead four sets of plots. The CNTRL CRF plot is closely matched to the EXPL CRF plot which observably show the fastest rates of learning. The EXPL PRF plot is slower to learn than the EXPL CRF plot but converges to close to 100% correct performance ($\mu =96.77$) by the final block of trials (each block represented is a mean over 50 simulation runs).

#### Extinction phase

**Fig. 10:**
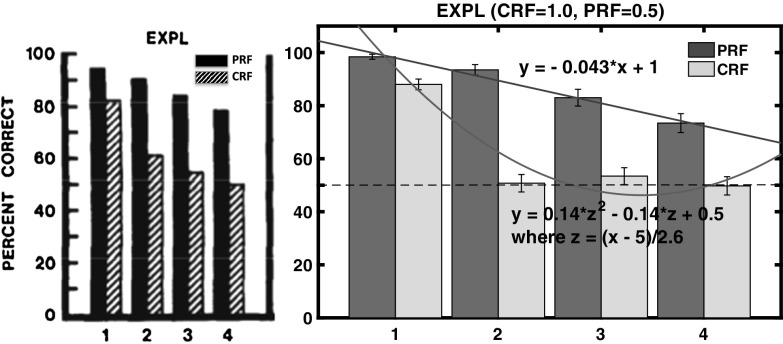
Comparison of the present theoretical predictions with Kruse and Overmier acquisition results. *Left* original experimental results due to Kruse and Overmier (1982), reprinted with permission. The x-axis label 1-4 consists of 4 blocks of 32 trials, blocks administered per day over 4 days. *Right* simulated results. The x-axis label 1–4 consists of blocks of 10 trials each. Key: *EXPL* experimental condition, which in the Kruse and Overmier investigation (and our simulated replication), represents the within-subjects condition; *PRF* partial reinforcement component/schedule; *CRF* continuous reinforcement component/schedule

The results of the extinction phase are displayed in Fig. 10. In Fig. 10 (left) the empirical data is shown of mean values of ‘correct’ behavioural choices (i.e. corresponding to same-choice per schedule in the acquisition phase). We show here only the data from the EXPL (CRF–PRF components) condition. No statistically significant differences in the control (PRF-only, CRF-only) conditions were found nor between conditions. Thus, *the experimenters found a within-subjects PREE for performance choice*.

In our simulations, the PRF trials can be observed to produce choices that are more resistant to extinction over trial blocks up to the point of extinction (CRF standard error bars overlap with random, i.e. 50% correct performance). Polynomial regression lines are plotted for both the PRF extinction (linear), and CRF extinction trajectories (quadratic), which appear to be consistent with appropriate representations also of the original empirical data. Consistent with the notion that the network requires more omissions of reinforcers in extinction in the probabilitistic (PRF) rather than unconditionally reinforcing (CRF) condition (Gallistel and Gibbon 2000; Nevin 2012), in CRF extinction of correct responses is rapid, whereas in PRF it is comparatively slow. Remaining unanswered, on this popular account of the PREE, is what determines the rate of extinction for the different schedules?

### Simulation of Svartdal (2008) within-subjects experiment

In Svartdal’s (2008) experiment, there are three conditions (within-subjects and two between-subjects conditions). The procedure is similar to that of Kruse and Overmier (1982) as described in the previous section (see Fig. 3). The key difference in this study is replacement of an unconditional (CRF) reinforcement schedule by a ‘high-density’ probability of reinforcement (0.8) condition while the low-density (PRF-equivalent) is set at 0.4 reinforcement probability (compared to 0.5 in Kruse and Overmier 1982).

The most important methodological difference between Svartdal’s set-up and that of Kruse and Overmier is that for each of the two predictive stimuli, the same response rule applies: produce the mirror-opposite of the two-button sequence presented on the monitor. Contrarily, in the Kruse and Overmier (1982) experiment each schedule has an *independent* task rule: if S1, choose R1; if S2, choose R2.

#### Acquisition phase

**Fig. 11:**
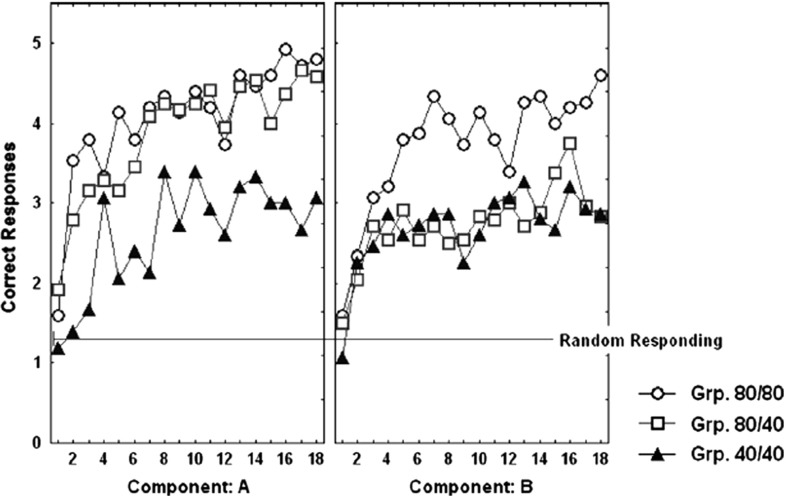
Acquisition learning correctness performance for original Svartdal (2008) data. *Left* Component A results for each of the three subject conditions. *Right* Component B results for each of the three conditions. Component A concerns stimulus 1 presentations and Component B stimulus 2 presentations. In within-subjects conditions, this results in density 80 (0.8 probability) and 40 (0.4 probability), for the respective components. The difference between Component A and B conditions entail consistent presentations of one of two predictive stimuli arbitrarily selected by the computer program at the beginning of the experiment for a given subject (reprinted with permission from Svartdal 2008). Plots are based on 18 blocks of 5 trials for each component

The results of the acquisition phase of the Svartdal (2008) experiments, over the three conditions, are shown in Fig. 11. It is observable that acquisition learning is faster in the ‘high-density’ schedule as compared to the ‘low-density’ schedule.^8^

**Fig. 12:**
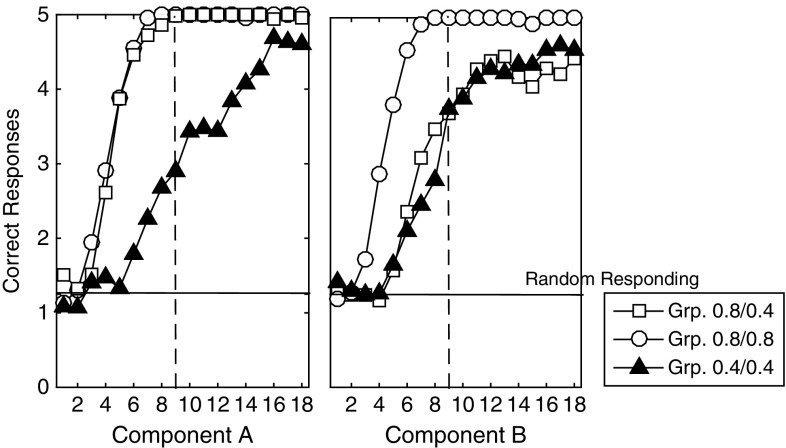
Acquisition learning simulated correctness performance. Correct responding means are shown over 18 blocks of 10 trials ($\approx$5 trials per component). Here, component A always involves presentation of stimulus 1, while component B always involves presentation of stimulus 2 at the beginning of each trial. The stimuli are binary numbers so not considered to bias the results. The *vertical dashed line* shows at block 9 values that might be compared to those of Svartdal’s by the final trial block and reflect an approximate 1.5${\times }$ faster learning rate of the high-density component

The simulations results are given in Fig. 12. Qualitatively, we have similar results, i.e. high-density schedules learn to cue correct responses faster than low-density schedules. Moreover, the increasing rate of correct performance (where over 5 evaluation trials random performance is set at 1.25, i.e. 0.25 probability, as for Svartdal 2008) in several components has not asymptotically converged (similar to the Svartdal acquisition data).

#### Extinction phase

**Fig. 13:**
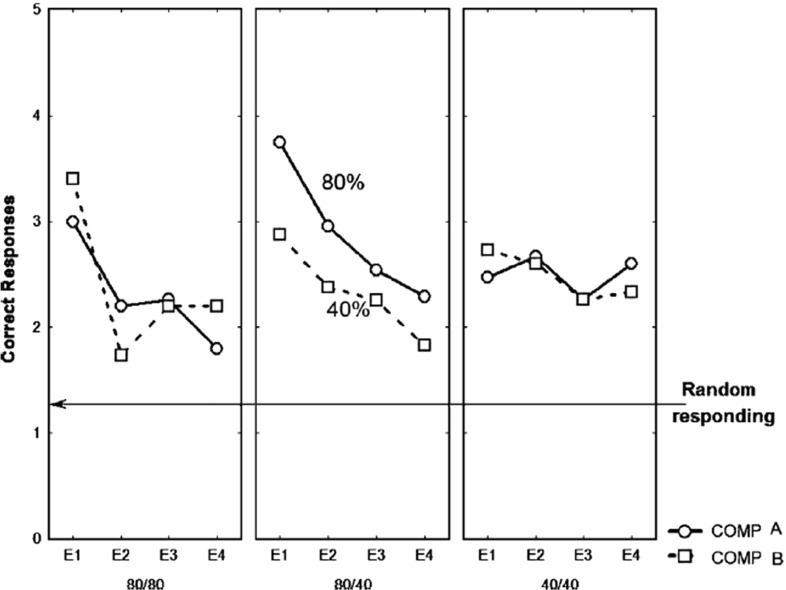
Extinction learning correctness performance for original Svartdal (2008) data. *Left* Component A (stimulus 1) results for each of the three subject conditions. *Middle* Results for subjects given a differential reinforcement probability stimulus schedule, i.e. 0.8 and 0.4 reinforcement probability for Component A (stimulus 1) and B (stimulus 2), respectively. *Right* Component B (stimulus 2) results for each of the three conditions. The difference between Component A and B conditions in the same-schedule conditions concerns stimulus presentation order. E1–E4 represent Extinction blocks 1–4 each consisting of 5 trials per component (reprinted with permission)

The results of the extinction phase for the three conditions of the Svartdal data are presented in Fig. 13. Of critical conceptual importance is the middle panel which shows the correctness performance in the multiple-schedule (high-density versus low-density), i.e. within-subjects, condition. The superior overall performance on the high-density schedule compared to the low density schedule is described as a RPREE. Svartdal reports that the findings of an ANOVA showed a group x trial block interaction effect. A further test showed a significant difference in performance of the high-density and the low-density in the multiple-schedule condition. This confirmed *Svartdal’s expectation of a within-subjects RPREE for performance choice*.

**Fig. 14:**
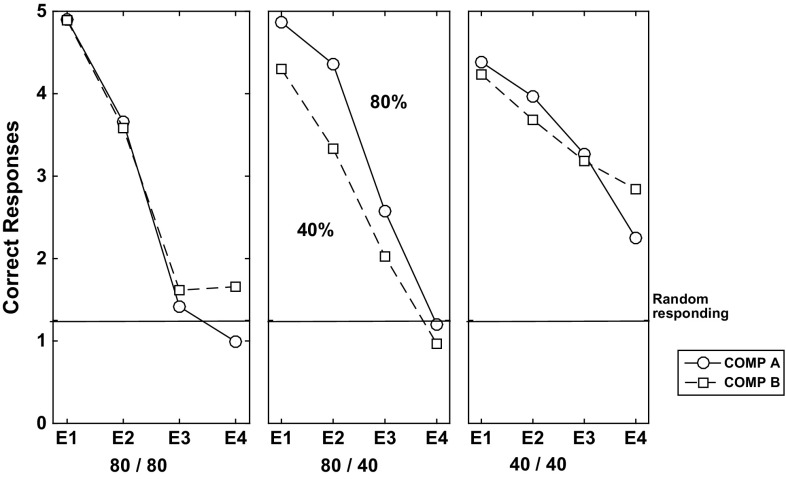
Extinction learning simulated correctness Performance. Here components A and B concern presentations for Stimuli A and B that are binary numbers. E1–E4 represent extinction blocks 1–4 each consisting of 5 trials

In our simulations, visualized in Fig. 14, it can be observed that, similar to Svartdal, the high-density schedule in the 80/40% rewarded (0.8/0.4 probability rewarded) condition produced higher choice correctness than did the low-density schedule. This was observed over fewer trials than for Svartdal (5 per block as compared to 10), which may owe to the learning rates chosen for our model. Nevertheless, the qualitative finding of an observable within-subjects RPREE was found. We considered only the second and third blocks for analysis, i.e. after *intertial transients* and before *absolute extinction* (affecting at least the 80/80 and 80/40 schedules by E4). We found the high-density component (M $=$ 0.641, SEM $=$ 0.023, 95% CI[0.596, 0.687]) scored higher than the low-density component (M $=$ 0.581, SEM $=$ 0.026, 95% CI[0.53, 0.632]). Using a mixed ANOVA we found significant effects for the within-subjects factor (*type of trial/schedule*; $F(1,147)=7.899, p<0.01$, $\eta _{p}^{2}=0.051$), the between-subjects factor (*experimental condition*; $F(2,147)=6.246, p<0.01$, $\eta _{p}^{2}=0.078$), and also an interaction effect: $F(2,147)=5.495, p<0.01, \eta _{p}^{2}=0.070$. We also conducted paired *t* tests to evaluate the within-subjects performance. In the multiple (0.8 vs. 0.4) schedule correct responding during extinction was found to be higher: $t(98)=2.5823, p<0.05$, 95% CI[0.0365, 0.2785]. This confirmed a within-subjects RPREE in simulation of Svartdal’s experiment. As expected, no significant differences were found when comparing 0.8 versus 0.8 ($t(98)=-0.2658$) and 0.4 versus 0.4 ($t(98)=0.5212$). So, consistent with the Svartdal data, our model predicts a within-subjects RPREE contrary to the Kruse and Overmier (empirical and simulated) results in the previous subsection. Why is this the case? We answer this question in the next section.

## Affective-ATP computation

In this section we investigate more closely the validity of the neurocomputational affective–associative description of the two sets of empirical data. We further wish to explain: (1) why partial reinforcement (or low-density) acquisition schedules extinguish faster than continuous reinforcement (or high-density) schedules, (2) why a PREE is found in the Kruse and Overmier within-subjects experiment but a RPREE found in the Svartdal experiment. We address these questions according to the following:

4.1. We discuss *stimulus classification by expected outcomes* (Urcuioli 2005, 2013) and evidence this phenomenon with a mechanistic analysis of our computational model.
4.2. We describe the results of lesions of the various connections of the tested model and, thereby, compare performance with a standard actor–critic model, and also an associative mediational theory (AMT) only model.
4.3. We carry out a parameter sensitivity analysis, which evaluates the range of learning rates permissible for our model to account for the two sets of data, as well as to be theoretically plausible in relation to other aspects of biological learning. We describe learning phenomena, related to the PREE phenomenon, that such a parameter range can account for.

We show in our comparative tests that the associative links that concern the full (affective) ATP model are both necessary and sufficient to qualitatively capture *all* the Kruse and Overmier, and the Svartdal, data. Simplifications of the model are thus insufficient to capture all the data.

### Stimulus classification by expected outcomes

**Fig. 15:**
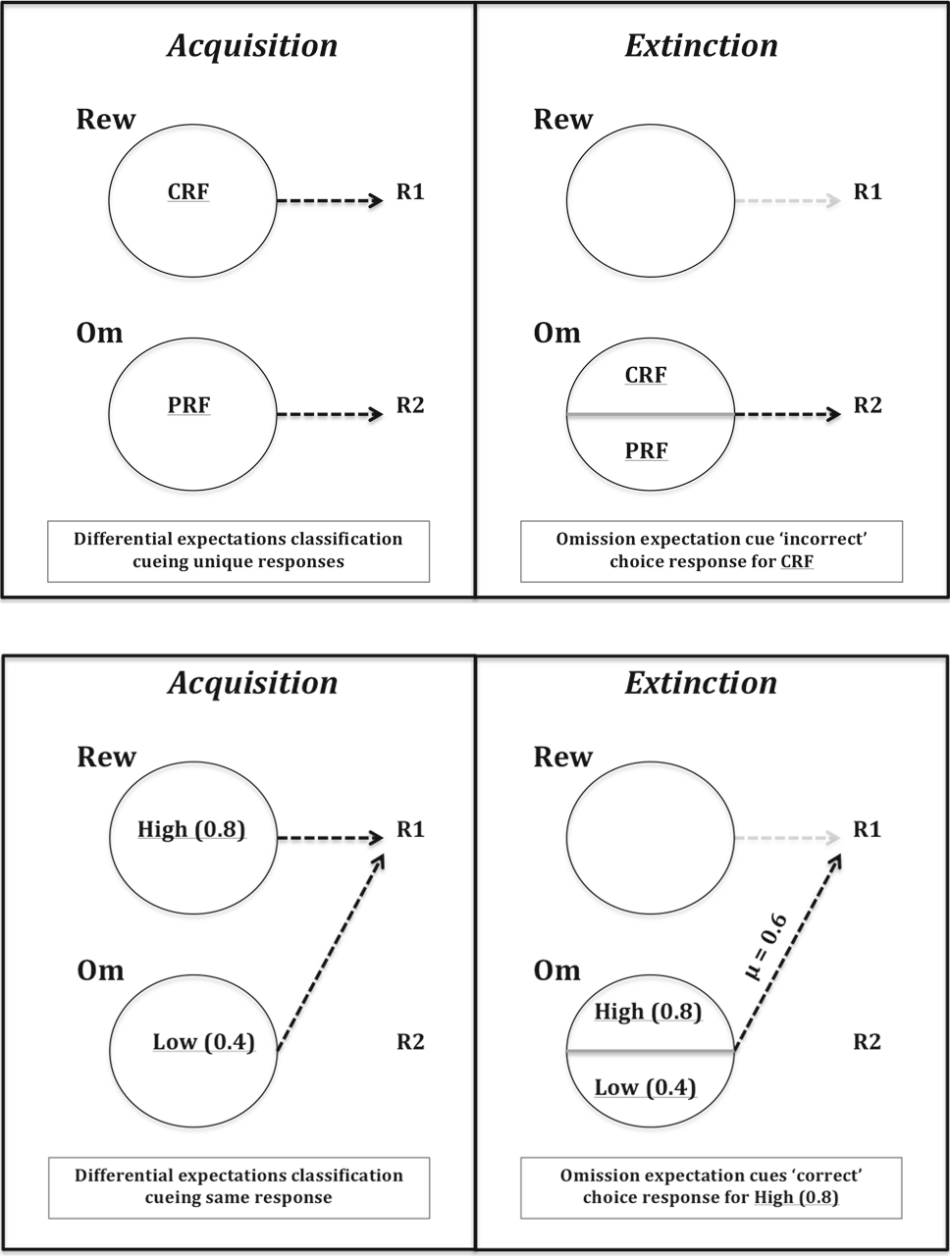
Acquisition–extinction transfer of control effects (Experimental condition). *Top* Kruse and Overmier (1982) transfer of control schema: during the acquisition phase, the CRF stimulus (CRF) is effectively classified by the differential expectation of reward, the PRF stimulus (PRF) is classified by the differential expectation of omission. These two differential expectations control/mediate differential responses. During the extinction phase CRF (as is still true for PRF) is now classified by Omission expectation and so cues the incorrect response (R2) due to the already learned omission–R2 association (Om2–R2 in our model). *Bottom* Svartdal (2008) transfer of control schema: during acquisition, similar to the Kruse and Overmier scenario, the high-density stimulus (high) is classified by reward while the low-density stimulus (low) is controlled by mission. However, unlike the Kruse and Overmier scenario, the differential expectations both control and mediate the same response (R1). Therefore, during the extinction phase, when high is now classified by omission expectation, it continues to mediate the ‘correct’ (i.e. same) response (R1)

Figure 15 schematically explains why the Kruse and Overmier (1982) experiment yields a PREE and the Svartdal (2008) experiment an RPREE for the within-subjects schedules. In line with Urcuioli (2005, 2013), we seek to explain these phenomena according to the ability of differential outcome expectations to classify discriminative stimuli and use these classifications to cue a subset of associated response options. We posit that differential outcome expectations classify stimuli associated with *reinforcement* schedules (CRF/PRF or High/Low probability) in both experiments under investigation in this article. The learned outcome classifications cue particular responses. In the case of Kruse and Overmier (1982) the response options (lever pressing behaviours) are unique and differentiated according to the two outcomes; in the case of Svartdal (2008) the response options (mouse button press task rules) are *not differentiated according to the two outcomes*. This means that during the extinction phase when both schedules are classified by omission expectations, in the Kruse and Overmier (1982) scenario the CRF stimulus will now cue the ‘incorrect’ choice (i.e. that mediated by the PRF stimulus in the acquisition phase) via the learned association of omission expectation and response 2 (R2). On the other hand, in Svartdal (2008) the omission classification of the High-density stimulus does not alter the response option—the same Omission-Response association is invoked. So, whereas in the CRF of Kruse and Overmier’s EXPL condition, incorrect responses are cued subsequent to *reward* $\rightarrow$ *omission classification switching*, in the Svartdal experiment *reward* $\rightarrow$ *omission switching* does not affect response selection.

**Fig. 16:**
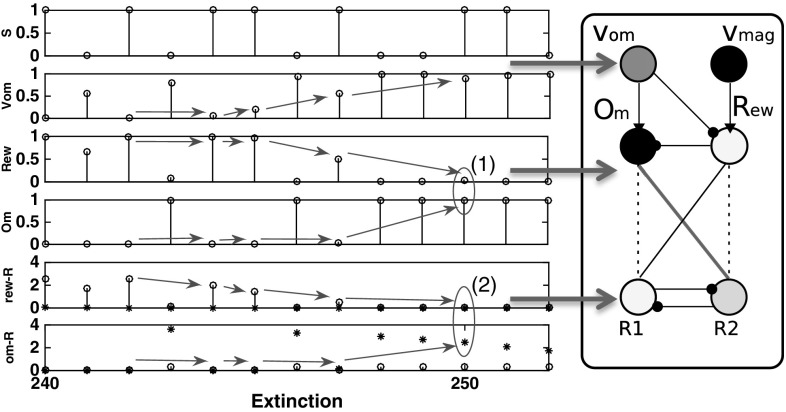
Learning of differential affective mediation of extinction responses, Kruse and Overmier EXPL condition case study. *Left* associative–affective network activations in the first 20 trials of the extinction phase. *Right* Network output activations (darker more active) corresponding to those encapsulated in the rectangle of the left figure. Dark connections between Om/Rew and R1/R2 indicate learned connections. *Dashed* connections indicate unlearned (but learnable) connections. Key: *S* stimuli, *1 values* Stimulus 1, *0 values* Stimulus 2; *Vom* Omission value node; *Rew* rew node; *Om* Om node; *rew-R* Rew node outputs to R1 (*open circles*) and R2 (*asterisks*)

**Fig. 17:**
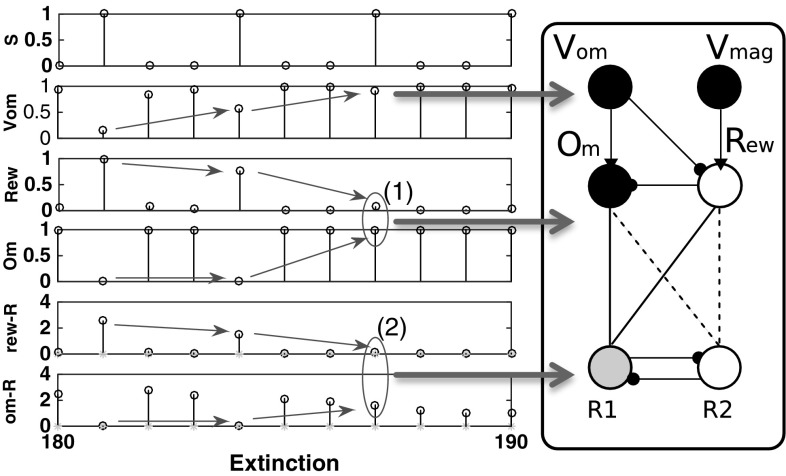
Learning of differential affective mediation of extinction responses, Svartdal 0.8/0.4 condition. *Left* Associative–affective network activations in the first 20 trials of the extinction phase. *Right* Network output activations (darker more active) corresponding to those encapsulated in the rectangle of the left figure. Key: *S* stimuli, *1 values* Stimulus 1, *0 values* Stimulus 2; *Vom* Omission value node; *Rew* rew node; *Om* Om node; *rew-R* rew node outputs to R1 (*open circles*) and R2 (*asterisks*)

In relation to the Kruse and Overmier (1982) simulated results, the above-discussed ‘differential response mediation’ benefits from computational explanation to comprehend this stimulus classification effect. During the Acquisition phase the network exhibits *differential* mediation of responding—Stimulus 1 is controlled by reward expectation and Stimulus 2 by omission expectation leading to respective response 1 and response 2 differential response mediation. During extinction (Fig. 16), however, the growing $V_\mathrm{om}$ node value (which represents the omission probability computed by the omission critic) leads to inhibition of the Rew node and activation of the Om node. Over trials the weighted output of the Rew node is unlearned, via the negative prediction error feedback, and the Om node assumes differential response control by virtue of having learned the Om–R2 association during the acquisition phase. So now, in agreement with Kruse and Overmier (1982) following CRF stimulus presentation omission expectancy cue[s] the PRF response. This means the omission expectation control increases the tendency for error (choosing the PRF-correct response) in the CRF condition when stimulus 1 is presented (see also Fig. 15, top). There is a *switch* from acquisition to extinction phases from reward (‘optimistic’) to omission (‘pessimistic’) expectancy control in the CRF component.

Further to the aforementioned *stimulus classification by expected outcome* explanation of PREE versus RPREE effects is why CRF extinction (in the PREE case) is so much more rapid than for PRF extinction (refer to Fig. 10). This depends on the learning rates of the S-E and E-R connections in the affective–associative two-process network. If the omission learning rate (S–E connection) is faster than the omission-response (E–R) learning rate, extinction will be apparently rapid—increasingly strong omission output will not be sufficiently offset by the weakening of the omission-response 2 weight. On the other hand, omission control tends to increase persistence (of the PRF-correct response) when stimulus 2 is presented. Similar to the CRF schedule effects of omission, fast omission learning rates render Om–R2 unlearning insufficient to rapidly extinguish this now redundant behaviour.

In sum, the much faster extinction rate of the CRF schedule, compared to the PRF schedule, is not adequately explained by the network requiring fewer reinforcement omissions in the extinction phase to be inconsistent with the schedule experienced in the acquisition phase. The CRF schedule, in the extinction phase, comes to be controlled by omission, which does not merely serve to unlearn CRF-acquisition responses (R1) but induces a strong bias towards PRF-controlled responses (R2) leading thereby to rapid decline in performance—a sort of counterfeit extinction. PRF extinction is slow because no such acquisition-extinction switch of control occurs. Noting that the above find is dependent on the relative learning-unlearning rates of S–E and E–R connections, we discuss more the theoretical implications of learning rates in 4.3.

For the Svartdal (2008) simulation results, critically, the two associations between differential outcome expectancies are made to the *same* response (R2). In our simulation, abstractly we represent Svartdal’s task rule to *produce the reverse responses to that displayed on the monitor* by a single response node whose random chance of being selected is one in four (assuming 4 possible pairings of responses if selected at random). Correct responses show an increasing proportion of correct responses during the acquisition phase that reduces towards chance levels (0.25) over extinction blocks. Figure 17 shows the output activations for the nodes of the affective–associative network in the extinction phase. During the acquisition phase, stimuli 1 and 2 are differentially classified by reward and omission expectation as for the Kruse and Overmier (1982) simulation. During extinction, the growing $V_\mathrm{om}$ node activation inhibits the Rew node and activation of the Om node. Gradually the Om node assumes differential response control for both stimuli as a result of the Om–R1 association learned during acquisition. This is the same as for the Kruse and Overmier simulation. However, in this case, S1 (cueing 0.8 reinforcement probability) continues to produce the ‘correct’ response when Om cues R1 since not only Om but also Rew were associated with R1 in the acquisition phase.

The above description is, therefore, consistent with a *stimulus classification by expected outcomes* explanation of partial reinforcement extinction effects. In the case of Svartdal’s experiments, omission and reward (acquisition) expectations differentially mediate responding but, owing to the nature of the experimental set-up, they cue the same response options. In the 0.8/0.4 condition, the reason for the RPREE is simply explicable according to the higher rate of reinforcement in the 0.8 schedule leading to superior performance, as a result of stronger weighted output via the retrospective (S–R) route (for brevity and space-saving results not shown), in the acquisition phase, than for the 0.4 schedule. This in turn leads to a lower rate of correct performance in the first trials of the extinction phase for the 0.4 component.^9^

### ATP lesioning comparison study

**Fig. 18:**
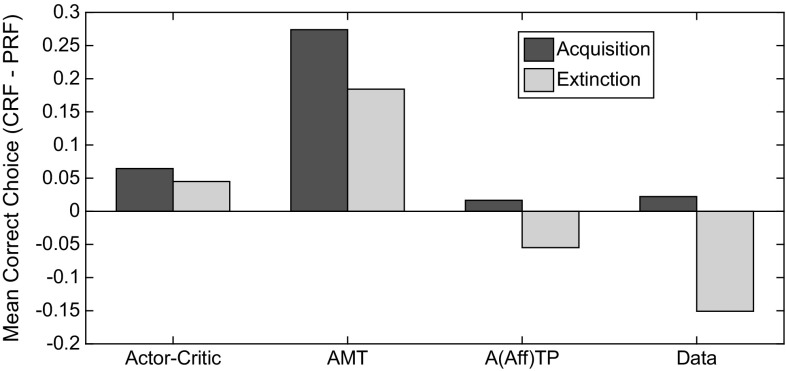
Comparison of models on Kruse and Overmier (1982) experiment. In this comparison, only the empirical data and the full ATP model produces CRF correct choice proportionate to PRF-correct choice ($\overline{{\hbox {CRF}}}-\overline{{\hbox {PRF}}}$)/($\overline{{\hbox {CRF}}}+\overline{{\hbox {PRF}}}$) transitioning from positive (in the acquisition phase) to negative (in the extinction phase). It is this transition that is characteristic of the PREE. Key: *AMT* associative mediation theory model, *A(Aff)TP* associative–affective two-process model, *Data* empirical data

**Fig. 19:**
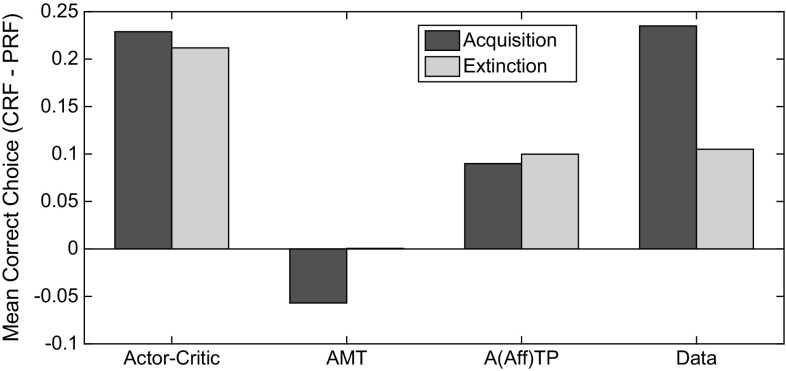
Comparison of models on Svartdal (2008) experiment. In this comparison, no model shows a PREE (again based on ($\overline{{\hbox {CRF}}}-\overline{{\hbox {PRF}}}$)/($\overline{{\hbox {CRF}}}+\overline{{\hbox {PRF}}}$) correct choice responses). The AMT-alone model shows a counterintuitive negative to positive (acquisition to extinction) transition in correct choice performance for CRF versus PRF. Key: *AMT* associative mediation theory model, *ATP = A(Aff)TP* associative–affective two-process model, *Data* empirical data. *Note*: Data smoothed by second-order polynomial over all data points obtained by Svartdal (2008)

In Figs. 18 and 19 are shown plots of acquisition–extinction correct choice performance, for representative single cases. As a measure of RPREE versus PREE performance we calculated: ($\overline{{\hbox {CRF}}}-\overline{{\hbox {PRF}}}$)/($\overline{{\hbox {CRF}}}+\overline{{\hbox {PRF}}}$), where $\overline{{\hbox {CRF}}}$ and $\overline{{\hbox {PRF}}}$ indicate mean correct performance for the CRF (high density) schedule, and for the PRF (low-density) schedule, respectively. This gives a minus value for a PREE and a positive value for an RPREE. This calculation was based on the last block of trials in both the Kruse and Overmier (1982) and Svartdal (2008) experiments (empirical data and simulated) for the Acquisition phase, and for the first 3 blocks of the Extinction phase for individual runs. We compared date from the following:

1. Actor–critic (prospective routed lesioned) model,
2. AMT (retrospective route lesioned) model,
3. (Affective-)ATP model,
4. Original empirical data.

The AMT (associative mediational theory, Kruse and Overmier 1982) model constitutes the affective–associative component of our full (affective-ATP) model and thereby lesions the S–R (retrospective route) component of the ATP. The actor–critic version of the model simply eliminates the connections between the critic and the actor inherent in the non-lesioned ATP model. All comparisons are made in the experimental (within-subjects) conditions where Kruse and Overmier and Svartdal found contradictory results, i.e. a PREE and reverse PREE (RPREE), respectively.

The Kruse and Overmier (1982) experiment and the full (affective-) ATP model are unique in producing a PREE over the acquisition–extinction transition (see Fig. 18).

For the actor–critic model, correct choice performance is dependent on learning the S–R connections (since E–R connections are lesioned). The strength of the connections in the Acquisition phase are determined by the reward prediction errors gating the Hebbian learning of connections between S representations and R response nodes. Therefore, for the CRF stimulus, the relevant S–R connection strength (here S1–R1) will converge to 1.0 as a function of learning rate, while for the PRF stimulus, connection strength (for S2–R2) will converge to 0.5. In extinction, the S–R connections are weakened by the negative reward prediction error generated by the Critic and it thus takes longer for the CRF (S1–R1) connection to extinguish as it is stronger in the acquisition phase. Hence, an RPREE result. For the ATP model, however, unlearning in the PRF component is offset by the increasing omission expectation value during extinction. This counteracts the effects of diminishing associations, i.e. E–R associations, through which response choice can be affectively mediated.

In Fig. 19, it can be seen that no model produces a PREE for Svartdal’s simulated experiment. The AMT model produces a compromised performance during the acquisition phase in the absence of scaffolding from the S–R route whereby initial S–R learning guards against the effects of initial erroneous E–R associations. ATP, actor–critic and the empirical data all show more standard RPREE findings, i.e. that the high-density component is implicated in slower choice correctness extinction than is the low-density component. The lack of a discriminative choice option in the Svartdal experiment (S1–R1 and S2–R1 are probabilistically rewarded but no other S–R pairs) means that outcome expectations cue the same response and thus do not provide additional information to that available to the S–R route. Thus, the E–R weighting is shared $((0.8 + 0.4) / 2)$ over the high and low densities and therefore acquisition and extinction rate are approximately the same for the two components. So, informationally, acquisition and extinction learning/unlearning here only requires an S–R route (actor–critic is sufficient).

In summary, following an associative (reinforcement learning) approach, both retrospective (S–R) and prospective (S–E–R) routes appear necessary to capture the modelled data. The retrospective route is critical for scaffolding the learning of the prospective route, while the prospective route is critical for the persistence of the partial reinforcement (PRF) schedule (relative to the continuous reinforcement schedule—CRF).

### ATP learning rate sensitivity analysis

**Fig. 20:**
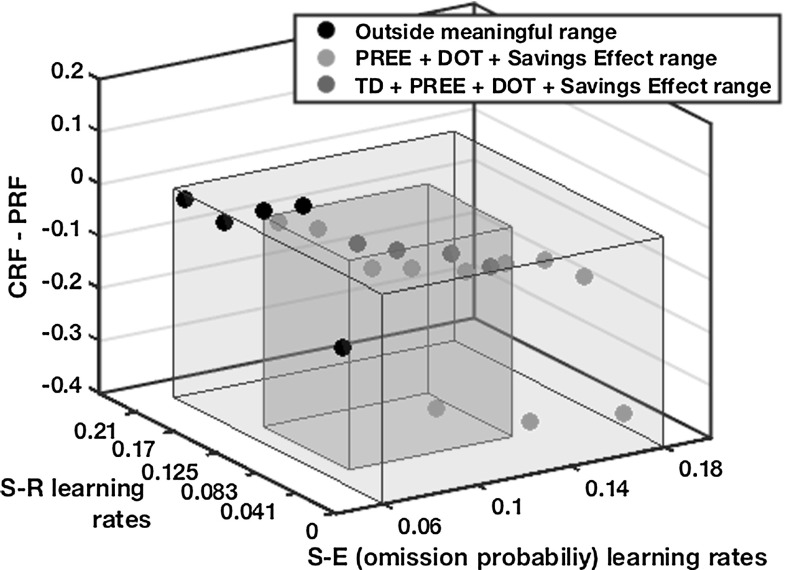
Learning rate sensitivity analysis for Kruse and Overmier (1982) data. The individual plots show ($\overline{{\hbox {CRF}}}-\overline{{\hbox {PRF}}}$) $\cdot$/($\overline{{\hbox {CRF}}}+\overline{{\hbox {PRF}}}$). *DOT* differential outcomes training modelled performance

The majority of the model parameters chosen for the experiments described are based on values consistent with TD learning theory and dynamic field theory. The critic values are given in Sect. 2.2.3 and actor values tabulated in “Appendix 6.3”.

Of critical importance to generating the partial reinforcement extinction effects found in the empirical data are those parameters concerning learning rates. Figure 20 shows, for the within-subjects condition of the Kruse and Overmier (1982) simulations, a scatter plot concerning parameterizations of S–R and S–E (omission value) learning rates as they map to PREE generation (CRF–PRF $<0$). Note, E–R and S–E (magnitude) learning rates are fixed at 0.06. The blue transparent box (with blue circles) encapsulates the range of values within which the PREE is achieved and the savings effect is also respected (where omission value updates must be higher than magnitude value updates for both effects). Within this range it is also possible to capture data from differential outcomes training scenarios (Lowe et al. 2014) where similar schedules are used as to the current simulation experiment but where outcomes can vary not just according to reward *omission probability* but also according to reward *magnitude*. The learning rates not encapsulated by the red box, however, might be considered implausible biologically and further untypical for TD learning simulations. Considering that the same range of parameter values for the Svartdal (2008) data always gives RPREE results, we can say that it is within this red box that we provide the most theoretically and biologically acceptable model, which simultaneously adheres to our hypothesis that omission probabilities may be approximated through neural activation.

We summarize the relationships between the different learning rates of the affective–associative two-process model for discriminable response tasks as follows:

1. S–E (omission) > E–R $$\rightarrow $$ PREE
2. S–E (omission) < E–R $$\rightarrow $$ RPREE
3. S–R < E–R $$\not \rightarrow $$ RPREE

Point (1) occurs because during the Extinction phase, if S–E learning is faster than E–R learning, absolute omission will soon be predicted and there will be no prediction error remaining on subsequent trials with which to unlearn the E–R weights. This means that as the omission (Om node) expectation controls responding of both PRF and CRF they will provide weighted output to the previously learned E–R response (for PRF) in the acquisition phase. Point (2) entails an RPREE since the E–R weights can be unlearned, through omission prediction error updates, before omission expectation probability of 1.0 is learned. Thus, while, as for Point (1), omission expectation controls responding for both PRF and CRF stimuli, there will cease to be E–R weighted outputs and responding will tend to random selection instead as a function of the degree of learning of CRF and PRF in the acquisition phase, which for all S–R, E–R learning rates is higher for CRF. Finally, in the case of Point (3), it appears that generation of expected PREE (Kruse and Overmier 1982) and RPREE (Svartdal 2008) results are not especially sensitive to particular S–R learning rates. Nevertheless, we have seen from our lesioning analysis that setting the S–R rate to zero prohibits the generation of appropriate effects in both experiments.

Thus, we conclude that so long as Point 1 is adhered to, that S–R learning rates are greater than zero, and that the full affective–associative two-process model with both retrospective (S–R) and prospective (S–E–R) routes is employed, it is possible to generate the network performance for simulations-based replication of *both* Kruse and Overmier’s (1982) and Svartdal’s (2008) data. If the retrospective route (S–R) is insufficiently influential in early learning (for scaffolding learning), erroneous prospective influence (via E–R connections) may result while the ‘pessimistic’ and ‘optimistic’ affective XOR-like classifications are still being meta-learned (see Fig. 23, “Appendix 6.4”). Thereafter, continuous erroneous prospective bias in decision making will render the task rules (S–R associations) difficult to learn. If S–E (omission) does not update sufficiently quickly, relative to S–E (magnitude), the strong influence of omission representation, relevant to the ‘savings effect’, the PREE and other differential outcomes learning data, will not be possible. This bears some resemblance to the learning of value in the brain where orbitofrontal cortex inhibits amygdala output in a fast and flexible manner in relation to context change (Schoenbaum et al. 2007). Thus, these relative learning rates are essential to capture the interaction between retrospective and prospective processing routes that in turn captures the described empirical data.

## Discussion

In this paper, we have provided a neural-computational description of an affective–associative two-process account of partial reinforcement extinction effects which helps reconcile contradictory (PREE versus RPREE) findings in the literature (Kruse and Overmier 1982; Svartdal 2008). The neural-computational model explains both sets of findings according to the standard *generalization decrement* invocation (Nevin 2012)—the schedule with lower probability reinforcement for correct responding in the acquisition phase of the experiment resembles more the zero reinforcement for ‘correct’ responding in the extinction phase (lower generalization decrement). The use of a single reinforcing response option indicates that Svartdal’s modulation hypothesis explanation for his within-subjects RPREE findings may owe to a *shared generalization decrement* over the two reinforcement schedules as a result of the differential mediators both cueing the same response choice. In this case, the generalization decrement for each reinforcement schedule (high vs. low density) in Svartdal’s within- subjects condition uses the average weighted response of the schedules for learning (acquisition)/unlearning (extinction) of the single rewarded response.

In the remainder of this section, we will discuss the following issues: (5.1) *Predictions of the Model*, (5.2) *Critique of the Modelling and Simulation Approach*, (5.3) *Alternatives to the ATP Hypothesis*.

### Predictions of the model

A main premise of this work is that Svartdal’s (2008) experiment introduces only one viable (rewarding) response option—mouse-press reversal of sequence presented on screen—in response to differentially (probabilistically) rewarding stimuli. We suggest that this fails to tap into the same phenomenon uncovered by Kruse and Overmier (1982)—associative mediational theory (AMT), which assumes omission, and reward, based expectancy control over *differential responses*. On this basis, while the AMT, when embedded within an associative two-process architecture, can explain both Svartdal’s (2008) data and that of the Kruse and Overmier (1982) experiment, Svartdal’s modulation hypothesis (Svartdal 2000, 2008), on the other hand, describes his own findings, but not those of Kruse and Overmier (1982). We have suggested, consistent with Urcuioli (2005, 2008, 2013), that stimuli predictive of differential *affective* outcomes can approximately classify such stimuli, which then bring to bear on existing associated responses. This particular effect is even more pronounced in Pavlovian-instrumental transfer (or ‘transfer-of-control’) set-ups (Urcuioli 2005), which demonstrate how novel stimuli previously only associated with differential outcomes but not responses may, without any requirement for learning, cue appropriate responses for reward retrieval. This is the result of the prospective route using existing associations concerning S–E (from the Pavlovian phase) and E–R (from a previous instrumental phase).

Our model, beyond the original AMT, makes the prediction that omission and reward representations have mutually inhibitory effects that promote an XOR-like classification of stimuli. AMT remains silent on this point (Overmier, personal communication). Such mutual inhibition permits cleaner expectancy-based response control. In our model, in the absence of this mutual inhibition, the competition between the two representations renders differential response control challenging. For example, in the Kruse and Overmier (1982) PRF set-up, omission and reward representations, without mutual inhibition, would both approximate 0.5 and no such differential response mediation (and thus PREE) should be possible. Further, our model predicts, that in an adaptation of the Svartdal (2008) experiment that uses two differential response options (i.e. different rules for each differentially rewarded stimulus), a PREE is possible, though owing to the relatively small difference between the probabilistic schedules (0.8 vs. 0.4 reward probability), the ability of the network to differentially classify stimuli might be challenging and requires empirical validation. In general, the bigger the difference between the two probabilistic schedules, the bigger the PREE effect is likely (Urcuioli 1990).

Our model also predicts that the ISI (inter-stimulus interval), if sufficiently reduced, would fail to produce a PREE. Omission expectancies in our model, as they bring to bear on response choice mediation, build nonlinearly over the ISI. Very short ISIs, therefore, should make expectancy mediation of responding more challenging and the retrospective route would become dominant.

Our focus has been on a neural-dynamic reinforcement learning computational description. We are currently investigating how well our model captures data according to differential outcomes training paradigms. Specifically, we are interested in *reward-based* differential outcomes training procedures—transfer of control—see Lowe et al. (2014), and also Lowe and Billing (2016), Lowe et al. (2016). The neural-dynamic (Amari-based) perspective of our model is thus used in a population coding capacity. This allows us to investigate existing hypotheses concerning spatial and temporal aspects of learning and decision making, e.g. regarding the neural-dynamic relation between prospective and retrospective topographically organized memory as it develops over an organism’s lifetime.

### Critique of the modelling and simulation approach

We have sought to validate a neural-computationally plausible model and, as such, have used neural-dynamic equations and a neural-anatomically plausible structure (actor–critic like) to frame our modelling approach. By modelling ATP using a neural-computational reinforcement learning approach, we can apprehend: (a) ATP’s neural-dynamic properties, (b) ATP according to a Markovian decision process, in relation to the animal/human learning and decision making data that are captured. Notwithstanding, a number of limitations of our model warrant further discussion. (1) Modelling temporal inputs: We have used the biologically unrealistic complete serial compound (CSC) temporal stimulus representation. This presupposes (i) a constant non-decaying relayed signal from external (predictive) stimulus onset to reinforcement onset, and (ii) leads to same-size negative prediction error signals as positive prediction error signals. Neither presupposition is tenable. An alternative model, of Ludvig et al. (2008, 2012) provides a potential solution to these problems. Microstimuli representations of the stimulus onset produce variably parameterized radial basis functions whose sum constitutes a decay from stimulus to reinforcer onset. This model also generates negative prediction errors more reflective of neurobiological reality, i.e. shallow and broad below-baseline activation as a result of expected reinforcer omission, while preserving the standardly found phasic positive prediction error signal (at unexpected reinforcer onset). We aim to apply this model to our affective–associative two-process model to further test the plausibility of our neural-computational approach. (2) Modelling magnitude of reinforcement: We implemented only non-negative learning of the magnitude critic. This reflects the suggestion that what is unlearned is not so much reward presence and strength but rather its effects on behaviour (cf. Morén 2002). However, in another version of our model (Lowe et al. 2014) tested on a differential outcomes learning problem, we allow for a slow unlearning rate of magnitude in order to be able to compute differential magnitudes as objective stimulus value changes.

Furthermore, our simulated approach made several assumptions that might be criticized. Firstly, we assumed that Svartdal’s response options could be abstractly considered as: (1) *produce the opposite response to that observed on the computer monitor*—the correct choice, (2) *produce a random response based on the permutations of the button pressing*—incorrect choice. In the case of following option (2), by a 1/4 chance, the correct response will be *eventually* discovered. Further, this random selection ‘strategy’ might be a reasonable assumption when considered over the uninformed population of subjects, i.e. an initial trial-and-error approach is utilized until the response rules are discovered.

Secondly, in the Kruse and Overmier (1982) simulation set-up (“Appendix 6.1”) we used the same inter-stimulus interval (22 processing steps) as for Svartdal (2008) when in reality the former was considerably longer (3 s) than the latter (0.7 s). We made this design decision to facilitate comparison of the data but with a longer delay for the Kruse and Overmier simulation replication, we might expect slower learning but similar overall results.

**Fig. 21:**
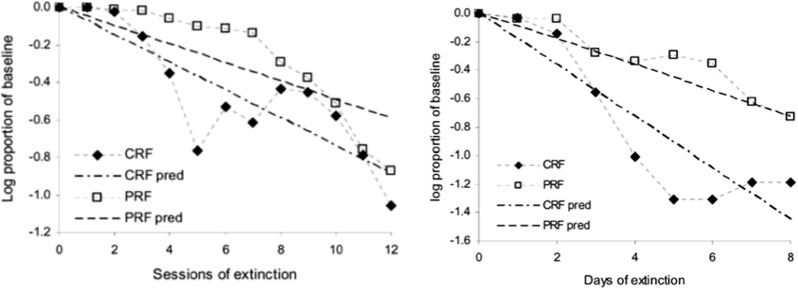
Behavioural momentum theory (BMT) modelling of Nevin and Grace (2005b) of empirical PREE findings. The pred (prediction) linear plots provide the BMT model of two choice discrimination task. A PREE is evident when considering the log proportions of the pre-existing correct responses (*baseline*). *Left* Discrete trial choice discrimination task Nevin and Grace (2005b). *Right* Discrete trial rate-based task Rescorla (1999). Reprinted with permission

Finally, it may be argued that comparing experiments using rats and humans does not provide a good basis for discerning shared neural-computational mechanisms, particularly where the prefrontal cortex is concerned. However, the PFC-relevant areas we have postulated—orbitofrontal cortex and medial PFC—are considered shared, as agranular regions, across all mammals (Passingham and Wise 2012). We might expect differences in performance of animals where more complex tasks and task rules are required to be learned as other regions of cortex are recruited, but the basic outcome–expectancy-associative mechanisms may be common across mammalian species.

### Alternatives to the ATP hypothesis

Svartdal interpreted his within-subjects RPREE finding according to a modulation hypothesis: “If extinction performance under individual schedule components is modulated equally by their contexts, extinction persistence under the 80% component should be increased if the context was a 40% schedule, and persistence under the 40% schedule should be reduced by a corresponding magnitude if the context was an 80% schedule” (Svartdal 2008, p. 53). To demonstrate this phenomenon, Svartdal made further comparisons between the two components of the multiple-schedule condition and their concomitant density schedules for the single-schedule (0.8/0.8 and 0.4/0.4) conditions.

We present our simulations of Svartdal’s tests in “Appendix 6.5”. Notwithstanding our simulation-based confirmation of Svartdal’s hypothesis, that in within-subjects conditions high-density schedules give the appearance of ‘modulating up’ low-density schedules while low-density schedules give the appearance of ‘modulating down’ high-density schedules, the modulation hypothesis cannot explain the PREE result of Kruse and Overmier.

**Fig. 22:**
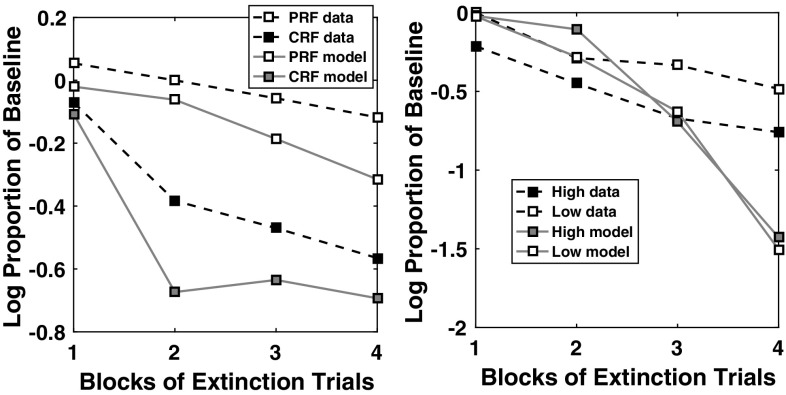
Modelling PREE versus RPREE empirical data according to rate of extinction. A PREE is still evident when considering the log proportions of the pre-existing correct responses (*baseline*) for both the Kruse and Overmier data and our model. The Svartdal data (and model results) do not support an RPREE in this case

**Fig. 23:**
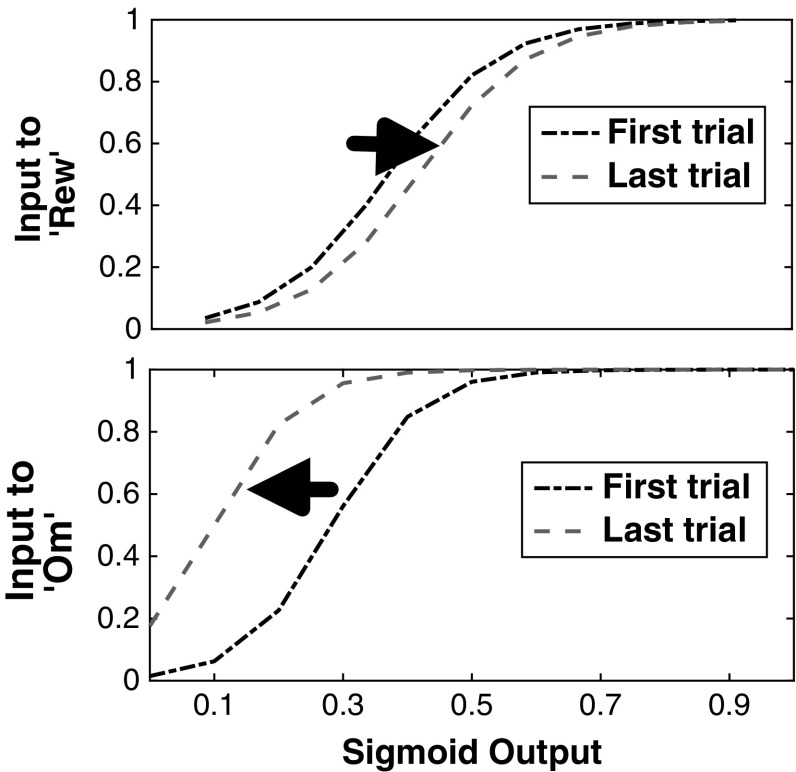
Neural-computational ‘classification’ in Svartdal simulation case study. *Top* Rew node sigmoid transfer function at the first trial of the extinction phase (*black line*) and last trial (*red line*). *Bottom* Om node sigmoid transfer function at the first trial of the extinction phase (*black line*) and last trial (*red line*) (colour figure online)

In Sect. 2.1. (see Fig. 1) we described two types of studies of the PREE—(1) rate-based, i.e. concerning an evaluation of the number of responses to stimuli predictive of a frequency of reinforcer presentations, (2) choice-based, i.e. concerning an evaluation of responses on discrete trials where reinforcement is presented in relation to choosing between *different* response options. Much research carried out on the PREE has focused on (1), but our interest has been on the decision-making (choice-based) aspect of the PREE. The within-subjects PREE has been found in such choice-based set-ups and explained according to particular perspectives. One such is that the partial reinforcement schedule requires more omissions of reinforcement in the extinction phase than the continuous reinforcement schedule in order to unlearn reinforcement-based expectations (Gallistel and Gibbon 2000; Nevin 2012). In this case, evaluating extinction in the different reinforcement schedules according to rate of change of correct choice in choice discrimination tasks should yield differential linear gradients as a function of omission rate. Nevin’s *Behavioural Momentum Theory* (BMT) (Nevin and Grace 2005b; Nevin 2012) predicts this result. The BMT was used to account for data on discrete trials of pigeon choice discrimination (Nevin and Grace 2005b) or pecking response rate (Rescorla 1999) using a calculation of the log proportion of correct choice in the extinction phase (over blocks of trials) to the baseline achieved at the end of the Acquisition phase. The Nevin and Grace (2005b) experiment resembles most that of Kruse and Overmier (1982) with a choice discrimination task and a probabilistically rewarding schedule (1.0 vs. 0.25). It is arguable, however, that the linear fit of the BMT model to the data (see Fig. 21, left) insufficiently captures the mechanistic complexity of the PREE. In Fig. 22 the extinction data of the Kruse and Overmier (1982) and Svartdal (2008) experiments are expressed as log proportions to baseline along with our model’s predictions. The continuous reinforcement (CRF) data of Rescorla (1999), Nevin and Grace (2005b) and Kruse and Overmier (1982) can all be argued to yield a disproportionately high, nonlinear, rate of extinction by comparison to the partial reinforcement (PRF) schedules. Based on our affective-ATP computational modelling approach, we suggest that the nonlinearity owes to the effect of omission representation (‘pessimistic’ classification) for the CRF stimulus mediating a response option classified by that affective state, which previously was reinforced in the PRF schedule (in the acquisition phase). This imbues a bias for the ‘wrong’ response early in extinction in the CRF and may account for the sharp increase in extinction in the three aforementioned experiments (as visualized in Figs. 21 and 22). On the other hand, the PRF schedule is relatively persistent. This, we have suggested, owes to the omission representation increasing in strength and initially offsetting the effects of the no-longer-reinforced and diminishing connections between the PRF stimulus and response. When expressing Svartdal’s data logarithmically (Fig. 22, right), the data, and our modelling thereof, provide evidence against a RPREE.

The differences in the extinction rates of the two schedules (high density versus low density) is slight, and we have suggested that this (relative non-difference) owes to the single response (choice) option being associated with the (increasing) omission representation during extinction tending, at a similar rate, to random responses in both schedules. We suggest that, at least as concerns choice discrimination tasks, the (affective)-ATP model is of greater explanatory value than both the BMT and Svartdal’s modulation hypothesis.

**Table 1:** Kruse and Overmier (1982) experimental versus simulations-based set-up

	Kruse and Overmier (1982)	Simulation
Subjects	6 rodents for each of the 3 conditions	50 simulations with different initial random seeds
Apparatus	Three colbourn modular rodent cages	MATLAB program adapting Cosivina Neural-dynamic software framework (http://roboticsschool.ini.rub.de/software.php)
External stimuli	Stimulus 1 $=$ sonalert tone, Stimulus 2 $=$ clicker	Binary valued Stimulus 1 and Stimulus 2
Pre-training	Yes (see paper for details)	No
Acquisition learning trials	40 (blocks) $\times$ 32 trials	10 (blocks) $\times$ 24 trials
Extinction trials	4 (blocks) $\times$ 32 trials	10 (blocks) $\times$ 4 trials
Inter-trial interval (ITI)	30 s	No ITI, neural activation reset at the end of the trial
Inter-stimulus interval (ISI)	3 s	22 processing steps (stimulus outcome)
Response requirement	10 presses of the correct lever (alternative lever retracted after initial press)	A single response selection
Response reward	CRF $=$ 1 pellet for correct response (10 presses) per trial; PRF $=$ 1 pellet for correct response (10 presses) at probability $=$ 0.5	CRF $=$ score 1 for correct response per trial; PRF $=$ score 1 for correct response at probability = 0.5
Between-subjects design	PRF-only condition, CRF-only condition	PRF-only condition, CRF-only condition
Trial ordering	(1) Trial transition probability 0.656; (2) No more than 3 successive trials of the same type could occur (for CRF and PRF types	(1) Trial transition probability pseudo-random; (2) No more than 3 successive trials of the same type could occur (for CRF and PRF types)

**Table 2:** Svartdal (2008) experimental versus simulations-based set-up

	Svartdal (2008)	Simulation
Subjects	56 students (male and female)	50 simulations with different initial random seeds
Apparatus	(1) A vertical metal console (33 $\times$ 33 cm), (2) two transluminated ’stimulus’ keys on a table in front of the console, (3) two push button ‘response’ keys on table, (4) sound attenuated room	MATLAB program adapting Cosivina NeuralGdynamic software framework (http://roboticsschool.ini.rub.de/software.php)
External stimuli	Stim. 1 $=$ red light, Stim. 2 $=$ green light	Binary valued Stimulus 1 and Stimulus 2
Pre-training	No	No
Acquisition learning trials	180 trials	180 trials
Extinction trials	40 trials	40 trials
Inter-trial interval (ITI)	3 s	No ITI, neural activation reset at the end of trial
Inter-stimulus interval (ISI)	0.7 s	22 processing steps (stimulus outcome)
Response requirement	To choose opposite sequence from that presented by the computer on lamps on the vertical console. 4 possible response contingencies: LL, LR, RL, RR (wrt push button positions). So 1/4 correct chance over blocks of 5 trials	A single response selection.
Response reward	Rewards $=$ score of 1 at probability: High dens $=$ 0.8 Low dens $=$ 0.4	Rewards $=$ score of 1 at probability: High dens $=$ 0.8 Low dens $=$ 0.4
Between-subjects design	LOW: S1 & S2$\rightarrow$Low dens $=$ 0.4; HIGH: S1 & S2$\rightarrow$High dens $=$ 0.8.	LOW: S1 & S2: Low dens $=$ 0.4; HIGH: S1 & S2: High dens $=$ 0.8
Trial ordering	Random presentation of S1 or S2	(1) Trial transition probability pseudo-random; (2) No more than 3 successive trials of the same type could occur (for CRF and PRF types)

In contrast, the RPREE phenomenon has been more typically found in rate-based experiments (Flora and Pavlik 1990; Nevin and Grace 2000; Nevin 2012) where the same response (non-choice discriminant) is typically required of the individual and rate of response is measured in accordance to number of reinforcers presented.

### Conclusion

In this article, we demonstrated neurocomputationally how affective–associative two-process theory is consistent with the within-subjects experimental findings of Kruse and Overmier (1982) and Svartdal (2008) who obtained contradicting PREE and RPREE results, respectively. We posited that critical to this contradiction is the lack of use of differential response choice options in the Svartdal (2008) experiment. Addressing the aims of our modelling approach put forward in the Introduction section, we mechanistically (and theoretically) described how a chief property of ATP theory—*stimulus classification by differential outcome expectancies*—may underly both sets of results. The modulation hypothesis of Svartdal (2008), confirmed by our simulations results, was explained according to the amount of omission prediction *error*—analogous to a dopamine signal—available to unlearn expectancy–response (E–R) associations during the extinction phase. The modulation effect amounts to a *shared generalization decrement* effect, consistent with Nevin (2012), affecting differential schedules.

Classifying stimuli by differential reward expectations serves little function if it cannot be put to some cognitive-behavioural end. One such end would concern (pessimistic) omission expectation that may motivate organisms to monitor environmental detail so as to find the stimuli complex that reduces uncertainty in prediction (Mackintosh 1971). Another use of such classification is to associate affective outcomes with a particular repertoire of adaptive responses. This accords with the somatic marker hypothesis of Damasio (1999). Somatic markers are affective states that can adaptively constrain action selection under conditions of uncertainty (e.g. when task rules are not well understood). In the *stimulus classification by expected outcomes* perspective, the affective states (reward, omission) generated in partial reinforcement experimental set-ups, classify particular stimuli by differential affective states that are then able to differentially cue choice responses. This particular property of these differential affective states, similar to somatic markers, is of most relevance when multiple response options can be associated with each affective state (cf. Urcuioli 2013).

## Figures and Tables

**Fig. 24:**
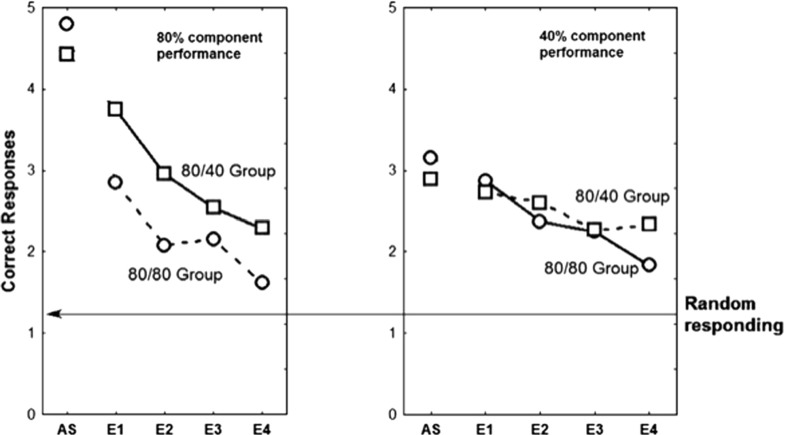
Comparison of multiple-schedule components to single-schedule concomitants for original Svartdal (2008) data. *Left* The 80% component of the 80/40 (multiple-schedule component) is compared to the 80/80 group Component A. *Right* The 40% component of the 80/40 group is compared to the 40/40 group (note, error in original labelling of Svartdal (2008) Component B. *AS* acquisition state, *E1–E4* extinction performance over four blocks of 10 trials. Reprinted with permission

**Fig. 25:**
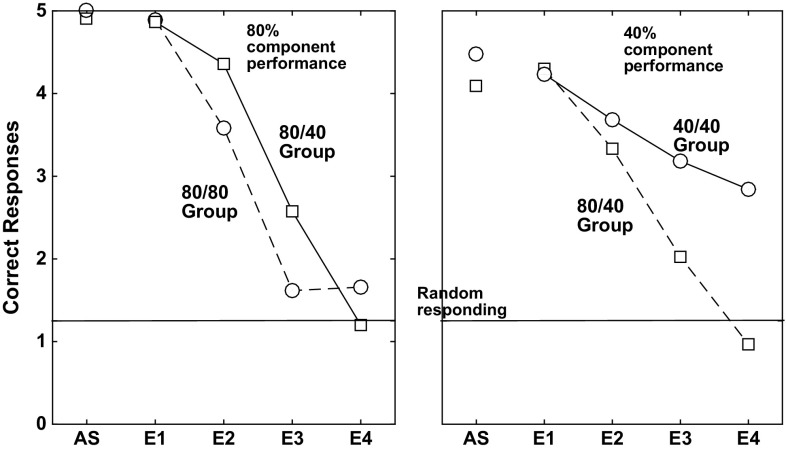
Simulation-based comparison of multiple-schedule components to single-schedule concomitants. *Left* As for Svartdal (2008), the 80% component of the 80/40 schedule is compared to the 80/80 schedule component A. *Right* The 40% component of the 80/40 schedule is compared to the 40/40 schedule component B

## Appendix

### Experimental versus simulation set-ups

Tables 1 and 2 compare the experimental set-ups of Kruse and Overmier (1982) and Svartdal (2008) to our simulations set-ups.

### A: glossary of key modelling terms

Table 3 provides a glossary of the key terms of our computational model.

**Table 3:** Glossary of key terms describing the full model in Fig. 8 and in Eqs. 1–12

$V_{e}(t)$	Learned value function (expectation) in [0,1] at time step *t* where $e\,\epsilon \,\{m,o\}$ and *m* stands for magnitude valuation of the stimulus, *o* stands for omission probability valuation of the stimulus. This is visualized in the nodes in the Critic in Fig. 8
$\theta _{e}(t)$	Gives the parameter (Critic weights) indexed by e that valuate the stimuli at each time step *t*. These weights are denoted by (1) on Fig. 8
$\delta _{m}(t)$	Is the prediction error generated by Critic that updates the $\theta _{m}(t)$ parameters of the magnitude Critic
$\delta _{o}(t)$	Is the prediction error generated by Critic that updates the $\theta _{o}(t)$ parameters of the omission Critic
$u_{r}(t)$	Stimulus/response option neural-dynamic variables indexed by $r\,\epsilon [1,R]$ where $R\,\epsilon \,\{S1, S2, R1, R2\}$
*Rew*(*t*)	Reward expectation (right-side blue node Fig. 8) classification of stimulus
*Om*(*t*)	Omission expectation (left-side blue node Fig. 8) classification of stimulus
$x_{s}(t)$	Meta-parameter that controls the slope and threshold of Rew and Om variables allowing for competition for stimulus classification
$\varOmega _{kl}$	Connection strength in [0, 1] between pre-synaptic node $k\,\epsilon \,\{Om,Rew\}$ and post-synaptic node $l\,\epsilon \,\{R1,R2\}$. See connections denoted by (2) on Fig. 8

### Free parameter values

Table 4 lists parameter values used over Eqs. (7–12) for our two simulated experiments. Note, the same parameterization is used for both experiments. The listed parameters concern standard values used in dynamic field theory modelling as well as parameterization of our meta-learning of affective classification functions (sigmoids). The parameter values given in Sect. 2.2.3 are standard and tested values for temporal difference learning.

The input term, from Eq. (7), is characterized by a standard dynamic field theory based formula: $I_{r=1|2}(t)$ = $C_{stim}$[$S_{cue}\varOmega _{kl}(t)$]$^{+}$ + $S_{tar}$ + $q\psi$, where $S_{cue}$ $\epsilon$ $\{$0,1$\}$ is the binary cue stimulus input and $S_{tar}$ $\epsilon$ $\{$1.25,4.5$\}$ is a bias term presented randomly for targets (choice options) per trial; $C_{stim}=3$ is a scaling term. See Eq. (12) for description of other terms.

Input $I_{r=3|4}(t)$ = $C_{exp}$[Om(t)$\varOmega _{n}(t)$ + Rew(t)$\varOmega _{n}(t)$] + $I_{r=1}$(t) + $I_{r=2}$(t) + $q\psi$, where $C_{exp}$=10 and $\varOmega _{n}(t)$ is the weighted input from Om, Rew nodes to n=1 (R1) and n=2 (R2), respectively. $\psi$ is a Gaussian noise term scaled by $q= 0.05$ and permits stochastic action selection in R nodes.

In Eq. (11), $x_s$ parameterizes the sigmoid update functions for (Om, Rew nodes) according to slope $x_{\beta _s}(t)$, $s=\{$1(Om),2(Rew)$\}$ and threshold $x_{th_s}(t)$, $s=\{$3(Om),4(Rew)$\}$. These are meta-parameters (Doya 2002) modulated by the prediction error feedback of the Omission Critic. $n_{s=1|2}\epsilon \{10,15\}$, $a_{s=1|2}\epsilon \{6,10\}$ give maximum and minimum values for $V_m$ and $V_o$ $\beta$ inputs, respectively. $n_{s=3|4}=0.4$, $a_{s=3|4}=0.1$ give maximum and minimum values for $V_m$ and $V_o$ *th* inputs, respectively.

**Table 4:** List of parameters not specified in main text

Parameter	Value
$\tau r= 1-4\,(decay\,constant)$	$\{3,3,5,5\}$
$hr = 1-4 \,(baseline\,activation)$	$\{-4,-4,-2,-2\}$
$Cr= 1-4 \,(self-excitation\,scaling)$	$\{10,10,1,1\}$
$\beta r= 1-4 \,(sigmoid\,gain)$	$\{5,5,4,4\}$
$Bom \,(sigmoid\,gain)$	20
$Thom \,(sigmoid\,threshold)$	0.2
$j=1-2 \,(meta-learning\,rates)$	$\{0.0667, 0.05\}$

### Meta-learning affective classification

In Fig. 23, is shown a representative example of the meta-learning of the sigmoidal transfer functions of the Rew node (Eq. 9, $V_m$ input), and the Om node (Eqs. 10/11, $V_o$ input). It can be seen that Rew ‘classification’ sensitivity becomes weaker (threshold drifts to the right) over extinction trials. This owes to the negative reward (positive omission) prediction error feedback to update the slope ($x_{\beta _\mathrm{vm}}$) and threshold ($x_{th_\mathrm{vm}}$) parameters. On the other hand, Om ‘classification’ sensitivity becomes stronger over extinction trials. This owes to the positive omission prediction feedback effect on increasing the slope steepness ($x_{\beta _\mathrm{vo}}$) and bringing the threshold ($x_{th_\mathrm{vo}}$) close to zero. Thus, we have a hypothesized meta-learning (Doya 2002) mechanism for permitting flexible classification by reward (acquisition/omission) outcome expectancy.

### Svartdal’s modulation hypothesis

Results of the analysis of Svartdal’s modulation hypothesis are displayed in Fig. 24. Svartdal found significant results for the 80% component, i.e. it showed greater persistence when the other component had a 40% schedule than when it had an 80% schedule which Svartdal attributed to the lower density component modulating *upwards* (making more resistant to extinction) the higher density component. He did not find a difference, however, regarding the 40% component being affected by an 80 or 40% alternative schedule though he predicted that in the 80/40 schedule the 80% component should modulate *downwards* (make less resistant to extinction) the lower-density (40%) component.

Our simulations for this pairwise comparison are visualized in Fig. 25. In paired *t* tests (evaluated over blocks 2 and 3 in the extinction phase) we found significant differences for both 80% (Fig. 25, left; $t(98)=-2.1896$) and 40% schedules (Fig. 25, right; $t(98)=3.6387$) where $p<0.05$. On this basis, our simulations actually provide results that are consistent with Svartdal’s modulation hypothesis for *both* high-density upward modulation (by the lower-density component) and by the low-density downward modulation (by the high-density component). The stronger simulated modulation effect of the 40% may reflect the higher acquisition performance value, in our simulation study, at which the extinction phase was initiated allowing for greater scope for differences in values until absolute extinction was achieved.

Notwithstanding our simulations-based confirmation of Svartdal’s hypothesis, we can explain these findings with recourse to *stimulus classification by differential outcome expectancies*. As Omission expectancy classifies both components of the low-density schedule, these components share a common association to R1. This means that they also share the amount of prediction error available, at the end of the Acquisition phase, to unlearn this association. This average amount of error is greater the higher the average density of the individual components. Thus, in the Svartdal (2008) experiment the average omission probabilities for the 0.8/0.8, 0.8/0.4, 0.4/0.4 conditions yield negative reward prediction error, averaged over the two components for each condition, of 0.8, 0.6 and 0.4, respectively, with which to unlearn responses.^10^ The extinction rate in these conditions is thus higher where the prediction errors are greater (more intense unlearning) which inversely reflects degree of reward expectation generalization from acquisition to extinction phases. This phenomenon can be likened to the *generalization decrement* effect: “Reinforcers, considered as stimuli, are part of the stimulus situation in which training occurs, and, when extinction begins, there is a smaller change in the overall stimulus situation after PRF than after CRF because the average reinforcer rate is lower” (Nevin and Grace 2000).

On the other hand, no such significant between-subjects effect was found in the Kruse and Overmier experiment (either in the empirical or simulated findings), which indicates that no explicit modulation mechanism exists.

## Footnotes

1 This is the delay between stimulus presentation and reinforcement presentation during which expectations can form.

2 We use the term target in reference to the dynamic programming term, i.e. the value to be approximated. When the target equals the value, no prediction error is generated.

3 Learning a reinforcing behaviour is faster in a reacquisition phase, following extinction, than in an initial acquisition phase.

4 We use the scaling term $\frac{\tau }{\Delta t}$ suggested in Doya (1996) rather than $\frac{1}{\Delta t}$ in Doya (2000) as the former allows for values of $\tau$ and $\Delta t$ greater than 1.

5 No schedule component was used for more than three consecutive trials.

6
Kruse and Overmier (1982), in the EXPL (or within-subjects) condition, show only results for an additional feedback condition which provides an additional cue for ‘correct’ responding, particularly relevant for the trials on which no reinforcer is presented.

7 Means were calculated over independent simulation runs (with different random seeds) over blocks of trials. See “Appendix” for details of simulations set-up.

8 Similar to the finding in the Kruse and Overmier (1982) acquisition phase, this result of *high reward probability* $\rightarrow$ *fast learning; low reward probability* $\rightarrow$ *slow learning* has been consistently borne out in the literature and is most simply explicable in terms of the increased frequency of reinforcement.

9 It is not clear in either the original Svartdal data (Fig. 13, middle panel) or the simulation data (Fig. 14, middle panel) that the gradient of extinction is lower for the 0.8 component.
